# Qing-Re-Qu-Shi formula improves clinical outcomes and is accompanied by gut microbiota and microbial metabolite remodeling in adults with moderate atopic dermatitis: a randomized placebo-controlled multi-omics trial

**DOI:** 10.3389/fmed.2026.1847003

**Published:** 2026-07-31

**Authors:** Yi Xiong, Peiyan Huang, Jihong Wang, Yuli Zhao, Jing Zhang, Dongming Wang, Huimin Zhang

**Affiliations:** 1 Department of Dermatology and Venereology, Anting Hospital of Jiading District, Shanghai, China; 2 Department of Dermatology, Shuguang Hospital Affiliated to Shanghai University of Traditional Chinese Medicine, Shanghai, China

**Keywords:** atopic dermatitis, gut microbiota, gut–skin axis, indole metabolites, metabolomics, Qing-Re-Qu-Shi formula, randomized controlled trial, short-chain fatty acids

## Abstract

**Background:**

The gut–skin axis is increasingly implicated in atopic dermatitis (AD), but randomized adult studies linking clinical response to paired gut microbiome and circulating metabolite profiling remain limited. We evaluated Qing-Re-Qu-Shi formula in adults with moderate AD and examined associated microbiome and serum metabolite changes.

**Methods:**

In this randomized, double-blind, placebo-controlled 12-week trial, 152 adults with moderate AD were assigned 1:1 to Qing-Re-Qu-Shi formula or placebo. Outcomes included Eczema Area and Severity Index (EASI), SCORAD, Patient-Oriented Eczema Measure (POEM), pruritus numerical rating scale (NRS), Dermatology Life Quality Index (DLQI), and responder rates. The primary clinical analysis followed the intention-to-treat principle. Paired fecal 16S rRNA sequencing and targeted serum metabolomics were performed in the biospecimen subset. Differential feature analyses adjusted for baseline level, age, and sex; *β* diversity was assessed using repeated-measures-aware PERMANOVA.

**Results:**

All randomized participants were included in clinical analyses; 124 contributed paired fecal and serum specimens, and 102 also had week-12 clinical data for cross-domain analyses. Compared with placebo, Qing-Re-Qu-Shi produced greater week-12 improvements in EASI (adjusted mean difference, −3.78; 95% CI, −4.54 to −3.01), SCORAD (−9.73; 95% CI, −11.54 to −7.93), POEM (−3.39; 95% CI, −4.06 to −2.73), pruritus NRS (−0.94; 95% CI, −1.15 to −0.72), and DLQI (−2.86; 95% CI, −3.47 to −2.26) (all *p* < 0.001). EASI-50 and EASI-75 responses were more frequent with Qing-Re-Qu-Shi. Shannon diversity changed little, whereas *β* diversity showed a small but significant group-by-time effect (*p* = 0.001, *R*^2^ = 0.016). Full-feature CLR analyses identified higher Blautia, Bifidobacterium, Lactobacillus, and Agathobacter and lower Escherichia/Shigella, Enterococcus, Prevotella, and Collinsella after false-discovery-rate correction. Serum metabolomics showed higher short-chain fatty acid and indole-related signals and lower kynurenine-related signals. Cross-domain correlations aligned these changes with greater symptom improvement.

**Conclusion:**

Qing-Re-Qu-Shi formula improved clinical severity and patient-reported outcomes in adults with moderate AD. These benefits were accompanied by selective gut microbial remodeling and circulating microbiota-related metabolite changes, supporting an associative link between clinical improvement and gut ecosystem remodeling rather than proving causality.

## 1 Introduction

Atopic dermatitis (AD) is a chronic, relapsing inflammatory skin disease with substantial global impact. Recent epidemiologic estimates suggest that AD affects both children and adults at meaningful scale worldwide, with roughly 2% of adults affected globally and a considerable cumulative disease burden across regions (1). Beyond visible skin lesions, AD imposes a broad clinical and humanistic burden through itch, sleep disturbance, impaired quality of life, psychosocial distress, and ongoing treatment demands, especially in patients with moderate and more persistent disease (2, 3).

The current understanding of AD pathogenesis emphasizes the interaction of epidermal barrier dysfunction, immune dysregulation, and microbial imbalance rather than a single dominant pathway. Barrier defects can facilitate allergen penetration and microbial perturbation, while type 2-predominant inflammation further amplifies barrier injury and chronic pruritus (4, 5). This multidimensional biology helps explain why many patients continue to experience fluctuating disease activity despite standard topical treatment and why biologically informed adjunctive strategies remain of interest (4).

Among these emerging directions, the gut–skin axis has attracted growing attention. Experimental and clinical observations suggest that alterations in gut microbial composition and function may influence systemic immunity, epithelial homeostasis, and inflammatory signaling relevant to skin disease. However, the literature in AD remains uneven. Much of the microbiome work has been cross-sectional, has focused on pediatric or mixed-age populations, or has relied on broad community-level descriptions without parallel metabolite profiling (5, 6). Importantly, a recent systematic review focused specifically on adults concluded that evidence for gut dysbiosis in adult AD is suggestive but still heterogeneous, underscoring the need for better-characterized prospective human studies in adult populations (6).

Several studies nevertheless provide a biologically coherent starting point. Reduced abundance of butyrate-associated or diversity-linked gut signatures has been described in AD, and disease severity has been reported to correlate inversely with gut microbial diversity and butyrate-producing bacteria in some cohorts (7, 8). In parallel, microbial metabolites are increasingly recognized as plausible effector molecules rather than passive readouts. Short-chain fatty acids, particularly butyrate, have anti-inflammatory and barrier-supportive effects in experimental settings (9). Tryptophan-derived indole metabolites are also of interest because they can engage the aryl hydrocarbon receptor (AhR), a pathway with relevance to epidermal differentiation, immune regulation, and cutaneous homeostasis (10, 11). These observations make circulating metabolite profiling a logical complement to taxonomic analysis when studying microbiome-linked changes in AD.

Clinical studies of microbiota-directed intervention in AD have yielded mixed results. Meta-analyses have suggested that probiotics may improve disease severity overall and may have measurable benefit in adults, but the literature is marked by heterogeneity in age groups, strains, formulations, duration, and endpoints (12, 13). Individual adult trials have reported improvement in AD-related symptoms with selected probiotic regimens, yet these studies have generally been limited in size and have not consistently integrated longitudinal microbiome and metabolite measurements with clinical outcomes (13, 14). As a result, an important gap remains between clinical efficacy data and mechanistic microbiome-related readouts in adult AD.

Herbal interventions are frequently used in East Asian clinical practice for chronic inflammatory skin disease, but microbiome-linked evidence from randomized adult AD trials remains limited. Qing-Re-Qu-Shi formula is used clinically with the therapeutic intent of reducing inflammatory activity and symptom burden, yet its potential association with gut microbial remodeling and microbial metabolite shifts has not been well characterized in a randomized, placebo-controlled setting. This is particularly relevant because biomarkers such as TARC are useful for tracking inflammatory activity in AD (15, 16), whereas microbiome-associated metabolites may help clarify whether symptom improvement is accompanied by measurable changes along the gut–skin axis (9–11).

The present study was therefore designed to evaluate Qing-Re-Qu-Shi formula in adults with moderate AD using a randomized, double-blind, placebo-controlled framework and an integrated multi-omics strategy. In addition to standard clinical endpoints, we assessed paired fecal 16S rRNA profiles and targeted serum metabolites related to short-chain fatty acid metabolism and tryptophan–kynurenine/indole pathways. We hypothesized that Qing-Re-Qu-Shi would improve clinical severity and that these clinical changes would be accompanied by selective shifts in gut microbial composition and microbial metabolites, rather than by a large, nonspecific increase in overall microbial diversity. By combining longitudinal symptom assessment with paired microbiome and metabolite measurements, this study aimed to provide a more clinically anchored view of the gut–skin axis in adult AD than is currently available in the literature.

## 2 Methods

### 2.1 Study design and ethics

This study was designed as a randomized, double-blind, placebo-controlled, 12-week clinical trial evaluating the clinical and gut microbiome-related effects of Qing-Re-Qu-Shi formula in adults with moderate atopic dermatitis. The protocol was approved by the Ethics Committee of Shuguang Hospital Affiliated to Shanghai University of Traditional Chinese Medicine (approval No. shtcm251322), and the trial was conducted in accordance with the Declaration of Helsinki and Good Clinical Practice principles. Written informed consent was obtained from all participants before enrollment. The trial was not prospectively registered, and the protocol is available from the corresponding authors on reasonable request.

### 2.2 Participants

Adults aged 18–65 years with a clinical diagnosis of atopic dermatitis were screened for eligibility. Diagnosis was established by dermatologists using standard clinical criteria based on the Hanifin and Rajka framework (17). Eligible participants were required to have moderate active disease at screening, defined as EASI 7.1–21.0 and SCORAD 25–50, together with investigator assessment. Key exclusion criteria included other major inflammatory skin diseases, recent systemic immunosuppressive or biologic treatment, recent use of antibiotics or probiotic preparations likely to substantially alter the gut microbiota, severe gastrointestinal disease, pregnancy or lactation, and other conditions judged by the investigators to interfere with trial participation or outcome interpretation. Standard diagnostic and severity instruments used in the study have been previously described and validated (18–21).

### 2.3 Randomization and masking

Participants were randomized in a 1:1 ratio to receive Qing-Re-Qu-Shi formula or placebo. The random allocation sequence was generated by an independent statistician using computer-based randomization and was implemented with concealed allocation. Participants, treating clinicians, investigators, and laboratory personnel remained blinded to treatment assignment throughout the trial. The placebo preparation consisted of inert granules based on dextrin and starch excipients with approved food-grade colorant and flavoring agents to mimic the appearance, smell, taste, packaging, and administration schedule of the active granules.

### 2.4 Intervention

Participants assigned to the active-treatment arm received Qing-Re-Qu-Shi formula in granule form for 12 weeks according to the study dosing schedule [one sachet (10 g) twice daily]. The formula was a fixed Qing-Re-Qu-Shi prescription prepared as granules under standardized manufacturing conditions; its constituent herbs and raw-herb equivalent amounts are provided in Supplementary Table S2. In traditional therapeutic terms, the prescription was designed to clear heat, remove dampness, cool blood, and relieve pruritus. Participants in the control arm received matched placebo for the same duration. Concomitant background skin care was standardized across groups, including routine emollient use and predefined rescue topical therapy when clinically required. Adherence was monitored at each visit by treatment reconciliation and participant interview.

### 2.5 Clinical and laboratory assessments

Clinical assessments were performed at baseline and at weeks 4, 8, and 12. The prespecified clinical efficacy endpoint for the present analysis was change in EASI from baseline to week 12. Secondary clinical outcomes included SCORAD, Patient-Oriented Eczema Measure (POEM), pruritus numerical rating scale (NRS), and Dermatology Life Quality Index (DLQI). EASI, SCORAD, POEM, and DLQI are established instruments for evaluating disease severity, patient-reported symptoms, and dermatology-related quality of life in atopic dermatitis (18–21). Responder outcomes were defined as achievement of EASI-50 and EASI-75 at week 12. Clinically meaningful EASI improvement was further explored using absolute EASI improvement thresholds of 3, 4, and 6.6 points. Transepidermal water loss (TEWL), total IgE, eosinophil count, and thymus and activation-regulated chemokine (TARC/CCL17) were measured at baseline and week 12 as exploratory barrier- and inflammation-related markers. TARC was included because prior studies have supported its value as a biomarker of disease activity and treatment monitoring in atopic dermatitis (16, 31). Rescue topical therapy was allowed according to the prespecified background-care plan, but rescue-use frequency was not quantitatively captured in the analysis dataset (Supplementary Table S11).

### 2.6 Biospecimen collection

Participants were asked to provide fecal samples and blood samples at baseline and week 12. Fecal samples were collected using sterile stool collection tubes, transported on ice, and stored at −80 °C until 16S rRNA sequencing. Blood samples were processed to obtain serum, and serum aliquots were stored at −80 °C until targeted metabolomics analysis. Fecal microbiome analyses were conducted in participants with paired baseline and week-12 stool specimens, whereas targeted serum metabolomics was performed using paired serum specimens available for the prespecified metabolite panel. A total of 124 participants had paired fecal specimens and paired serum specimens for omics analyses, and 102 had paired omics data and week-12 clinical outcome data for cross-domain association analyses linking fecal microbiome changes, serum metabolite changes, and clinical improvement (Supplementary Table S3). Clinical efficacy analyses followed the intention-to-treat principle and included all randomized participants.

### 2.7 16S rRNA gene sequencing and microbiome processing

Microbial genomic DNA was extracted from frozen stool using a stool DNA extraction kit with bead-beating incorporated to improve bacterial lysis, following the manufacturer’s instructions. The V3-V4 region of the 16S rRNA gene was amplified using dual-index primers 338F (5’-ACTCCTACGGGAGGCAGCAG-3′) and 806R (5’-GGACT ACHVGGGTWTCTAAT-3′). PCR reactions were performed in a 20 μL volume containing forward and reverse primers (200 nM each), template DNA (10 ng), nuclease-free water, and 5 × FastPfu DNA Polymerase (TransGen, Beijing, China). Cycling conditions were initial denaturation at 95 °C for 3 min, followed by 25 cycles of denaturation at 95 °C for 30 s, annealing at 55 °C for 30 s, and extension at 72 °C for 45 s, with final extension at 72 °C for 10 min. PCR products were resolved on a 2% agarose gel and quantified before sequencing. High-quality amplicons were sequenced on the Illumina NovaSeq PE250 platform (Illumina, San Diego, USA). Raw reads were merged, quality-filtered, and processed using QIIME 2 version 2021.8 (22). Amplicon sequence variants were generated with the DADA2 denoised-paired plugin. Features present in only one sample were filtered out, and taxonomy was assigned using a Naive Bayes classifier trained on the SILVA 138 99% reference database (23). Genera with mean relative abundance below 0.01% or prevalence below 25% were excluded from downstream feature testing. Zero values were handled using a pseudocount before centered log-ratio transformation. Shannon diversity was calculated as the principal alpha-diversity metric. Bray–Curtis dissimilarity was used for *β*-diversity analysis, followed by principal coordinates analysis (PCoA).

### 2.8 Targeted metabolomics

Targeted serum metabolomics focused on circulating microbiota-related metabolites selected *a priori* on biological grounds, including short-chain fatty acids (acetate, propionate, and butyrate), tryptophan, kynurenine, the kynurenine/tryptophan ratio, and indole-related metabolites such as indole-3-lactic acid, indole-3-propionic acid, and indole-3-aldehyde. Frozen serum specimens were thawed on ice and vortexed briefly. Serum aliquots were extracted using acetonitrile:methanol (1:4, v/v) containing isotope-labeled internal standards. After vortexing and centrifugation, the supernatant was collected for LC–MS/MS analysis.

Serum metabolite extracts were analyzed using an LC-ESI-MS/MS system consisting of an ExionLC AD UPLC system coupled to a QTRAP 6500 + mass spectrometer (AB Sciex). Chromatographic separation was performed on a Waters HSS T3 column (1.8 μm, 2.1 mm × 100 mm) maintained at 40 °C, with water and acetonitrile containing 0.1% formic acid as mobile phases. Scheduled multiple reaction monitoring was performed in triple-quadrupole mode using optimized precursor-product ion transitions, retention-time information, declustering potential, and collision energy. Quantification was based on chromatographic peak integration using MultiQuant software.

Pooled quality-control samples were injected after every ten analytical samples to monitor analytical stability and reproducibility. Metabolites with detection rates below 80% or quality-control coefficient of variation above 50% were excluded. Missing values were imputed using the K-nearest neighbor algorithm, and batch effects were corrected using support vector regression. Metabolite values were inspected for distributional properties before statistical analysis, and log2 transformation was applied where appropriate.

### 2.9 Statistical analysis

All statistical analyses were performed in R (4.5.3). Baseline characteristics were summarized by treatment group. In accordance with CONSORT-oriented reporting for randomized trials, baseline comparability was described using absolute standardized mean differences rather than significance testing. Baseline variables with SMD ≥ 0.25 were noted, and sensitivity ANCOVA models additionally adjusted for these imbalanced variables. Continuous clinical endpoints at week 12 were analyzed using analysis of covariance (ANCOVA), with the week-12 value as the dependent variable, treatment group as the main factor, and the corresponding baseline value as a covariate. Longitudinal trajectories for EASI and SCORAD across baseline, week 4, week 8, and week 12 were analyzed using linear mixed-effects models with fixed effects for treatment group, time point, and their interaction, and a participant-level random intercept. Responder outcomes (EASI-50 and EASI-75) were analyzed in the intention-to-treat population, with participants lacking week-12 assessment counted as non-responders. Missing continuous week-12 outcomes were further explored using last-observation-carried-forward, baseline-observation-carried-forward, multiple-imputation ANCOVA, and mixed-model repeated-measures sensitivity analyses. Adherence was summarized by treatment group and assessed in per-protocol and adherence-adjusted sensitivity models.

The sample size of 152 randomized participants (76 per group) was retained from the completed trial. A supplementary sample-size justification was provided based on the observed variability of EASI change and week-12 EASI. Using a two-sided alpha of 0.05, 80% power, and an expected between-group EASI difference of 3.0 points, the required sample size was lower than the actual randomized sample size under both observed-variance assumptions (Supplementary Table S7).

For alpha diversity, Shannon diversity was analyzed using linear mixed-effects models with fixed effects for treatment group, time point, and their interaction, with participant-level random intercepts. For *β* diversity, Bray-Curtis distance matrices were tested using repeated-measures-aware PERMANOVA with permutations restricted within participant ID; dispersion was assessed using PERMDISP, and within-subject Bray-Curtis distance from baseline to week 12 was compared between groups. Differential feature analyses for all tested microbial taxa and targeted metabolites compared between-group differences in change from baseline to week 12 while adjusting for baseline feature level, age, and sex. Taxa were analyzed after centered log-ratio transformation and metabolites after log2 transformation. For descriptive visualization, row-scaled heatmaps were generated from group-level mean values at each time point.

To explore cross-domain concordance, changes in fecal taxa and serum metabolites were correlated with improvement in EASI, pruritus NRS, and SCORAD using Spearman’s rank correlation among participants with paired omics data and observed week-12 clinical outcomes. Improvement scores were defined so that larger positive values indicated greater clinical improvement. Exploratory QRQS-arm responder analyses compared omics changes between EASI-50/EASI-75 responders and non-responders among completers. Exploratory mediation analyses were attempted for selected treatment-associated features, but were interpreted only as diagnostic because complete-case sample size was limited and indirect-effect confidence intervals crossed zero. Multiple testing was controlled using the Benjamini-Hochberg false discovery rate procedure. All tests were two-sided, and a false discovery rate-adjusted *p* value < 0.05 was considered statistically significant for high-dimensional feature analyses.

## 3 Results

### 3.1 Participant flow and analysis populations

**Figure 1:**
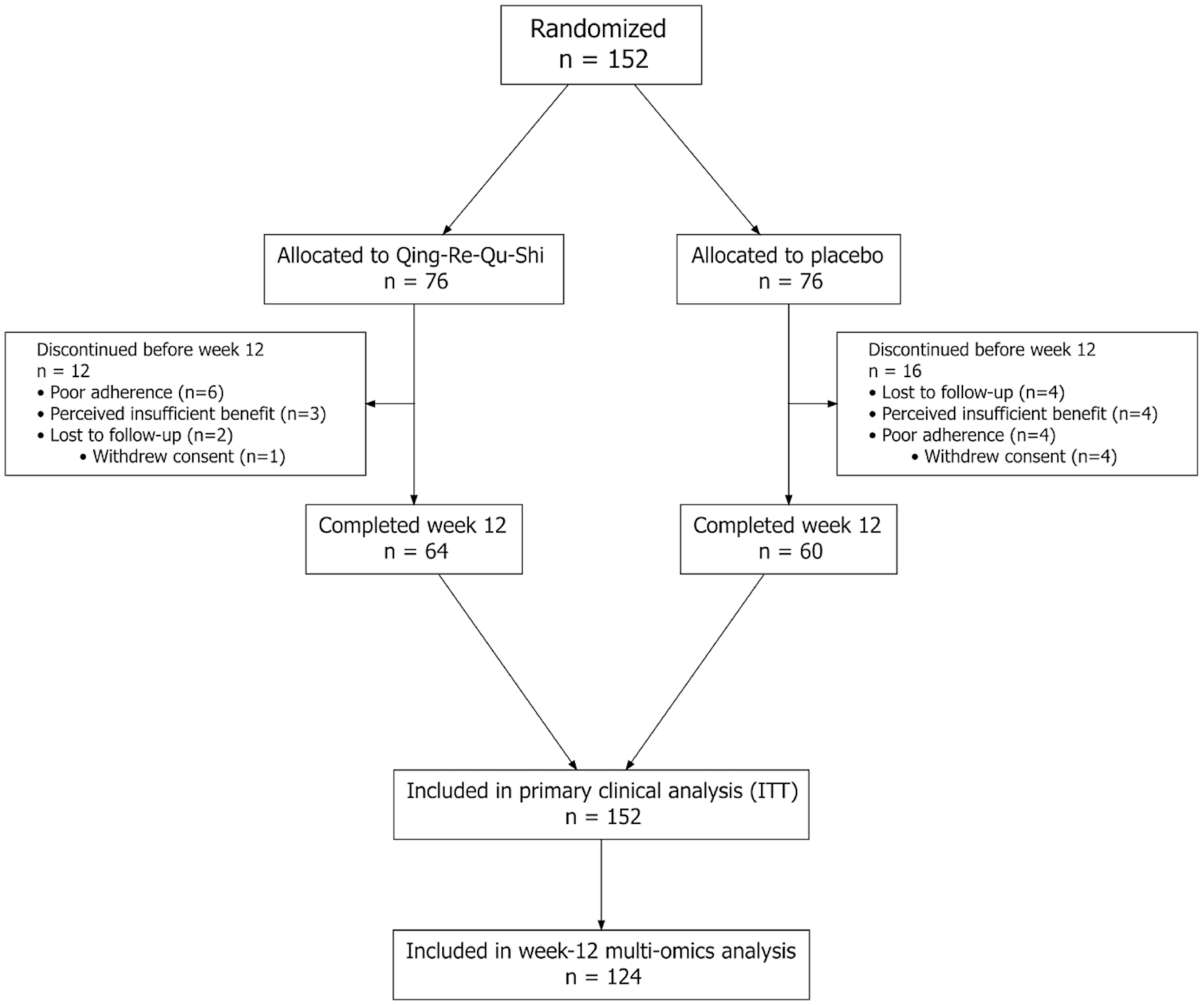
CONSORT flow diagram of participant progression through the randomized trial and the week-12 multi-omics analysis subset. Participants were randomized 1:1 to Qing-Re-Qu-Shi formula or placebo. The primary clinical analysis followed the intention-to-treat principle. Multi-omics analyses included 124 participants with paired fecal and serum specimens; cross-domain association analyses included 102 participants with paired omics data and observed week-12 clinical outcomes.

A total of 152 participants were randomized, with 76 assigned to Qing-Re-Qu-Shi formula and 76 to placebo (Figure 1). By week 12, 64 participants in the Qing-Re-Qu-Shi group and 60 in the placebo group had completed clinical follow-up. Paired baseline and week-12 fecal specimens and serum specimens were available for 124 participants. Because the clinical-completion set and paired-omics set were not completely identical, 102 participants had paired omics data and observed week-12 clinical outcome data and were included in cross-domain association analyses (Supplementary Table S3). The primary clinical analysis followed the intention-to-treat principle and therefore included all randomized participants.

Discontinuations occurred in both groups. In the Qing-Re-Qu-Shi arm, 12 participants discontinued before week 12, mainly because of poor adherence, perceived insufficient benefit, loss to follow-up, or withdrawal of consent. In the placebo arm, 16 participants discontinued before week 12 for similar reasons. No participant withdrew because of an adverse event or safety reason (Supplementary Table S25).

### 3.2 Baseline characteristics

Baseline clinical and demographic characteristics were broadly comparable between groups (Table 1). Most standardized mean differences were small, and all were below 0.30. Disease duration, asthma, and family history of atopy had SMDs greater than 0.25, indicating modest numerical imbalance (Supplementary Table S4). However, sensitivity models additionally adjusting for these variables yielded results consistent with the primary ANCOVA models (Supplementary Table S5). Baseline laboratory and barrier-related measures, including TEWL, total IgE, eosinophils, and TARC, were also well balanced.

**Table 1:** Baseline characteristics of randomized participants.

Characteristic	Placebo *N* = 76	Qing-Re-Qu-Shi formula *N* = 76	SMD
Age, years	32.8 ± 9.3	33.5 ± 10.5	0.075
Sex			0.053
Female	40 (53%)	38 (50%)	
Male	36 (47%)	38 (50%)	
BMI, kg/m^2^	23.4 ± 3.7	22.9 ± 3.5	0.143
Disease duration, years	6.1 (3.2, 9.6)	7.4 (4.7, 10.9)	0.269
Allergic rhinitis	34 (45%)	35 (46%)	0.026
Asthma	15 (20%)	8 (11%)	0.259
Family history of atopy	30 (39%)	20 (26%)	0.283
EASI at baseline	15.4 ± 3.8	15.7 ± 3.7	0.098
SCORAD at baseline	41.1 ± 8.2	42.7 ± 8.6	0.189
POEM at baseline	15.8 ± 3.6	16.2 ± 3.3	0.099
Pruritus NRS at baseline	6.6 ± 1.0	6.8 ± 1.1	0.205
DLQI at baseline	12.2 ± 3.5	13.0 ± 3.3	0.240
TEWL at baseline, g/m^2^/h	32.4 ± 8.1	33.8 ± 9.0	0.164
Total IgE at baseline, IU/mL	235.5 (147.4, 314.3)	230.9 (144.2, 376.4)	0.206
Eosinophils at baseline, ×10^9^/L	0.4 (0.3, 0.5)	0.4 (0.3, 0.5)	0.061
TARC at baseline, pg/mL	956.1 (714.5, 1,211.9)	936.4 (727.5, 1,329.1)	0.128

Data are presented as mean ± SD, median (IQR), or n (%), as appropriate. SMD indicates the absolute standardized mean difference. Variables with SMD ≥0.25 were considered to show modest numerical imbalance and were examined in sensitivity analyses.

### 3.3 Clinical efficacy over 12 weeks

**Figure 2:**
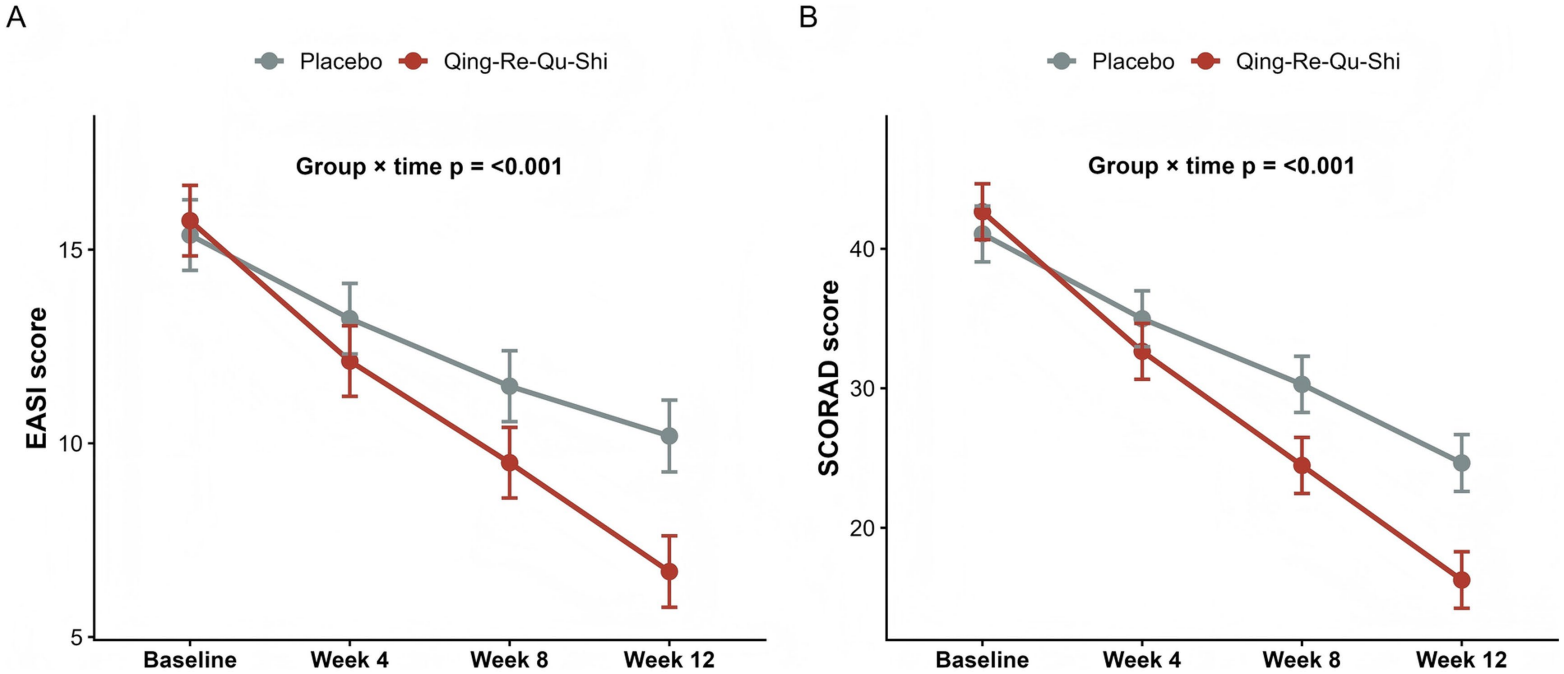
Longitudinal EASI and SCORAD trajectories by treatment group. **(A)** EASI score trajectories from baseline to week 12. **(B)** SCORAD score trajectories from baseline to week 12. Points indicate estimated marginal means and error bars indicate 95% confidence intervals from linear mixed-effects models with participant-level random intercepts.

Both groups improved over time, but the decline in disease severity was steeper in the Qing-Re-Qu-Shi group than in the placebo group (Figure 2). In linear mixed-effects models, the group × time interaction was significant for both EASI and SCORAD (both *p* < 0.001), indicating different symptom trajectories between treatment arms.

Among participants with week-12 assessment, mean EASI decreased from 15.4 ± 3.8 to 10.0 ± 4.3 in the placebo group and from 15.7 ± 3.7 to 6.5 ± 4.1 in the Qing-Re-Qu-Shi group (Table 2). The corresponding mean changes were −5.3 ± 1.9 and −9.1 ± 2.4, respectively. After adjustment for baseline score, the week-12 between-group difference favored Qing-Re-Qu-Shi, with an adjusted mean difference of −3.78 (95% CI -4.54 to −3.01; *p* < 0.001). This effect remained stable in sensitivity analyses for missing week-12 outcomes, including last-observation-carried-forward, baseline-observation-carried-forward, multiple-imputation ANCOVA, and mixed-model repeated-measures analyses (Supplementary Table S6).

**Table 2:** Changes in continuous clinical outcomes at week 12 by treatment group.

Endpoint	Placebo			Qing-Re-Qu-Shi formula			Adjusted mean difference at week 12 (95% CI)	*p* value
	Baseline	Week 12	Change	Baseline	Week 12	Change		
EASI	15.4 ± 3.8	10.0 ± 4.3	−5.3 ± 1.9	15.7 ± 3.7	6.5 ± 4.1	−9.1 ± 2.4	−3.78 (−4.54, −3.01)	<0.001
SCORAD	41.1 ± 8.2	24.1 ± 9.4	−16.6 ± 4.8	42.7 ± 8.6	15.9 ± 8.7	−26.5 ± 5.3	−9.73 (−11.54, −7.93)	<0.001
POEM	15.8 ± 3.6	10.3 ± 4.3	−5.5 ± 1.7	16.2 ± 3.3	7.2 ± 3.8	−8.9 ± 2.0	−3.39 (−4.06, −2.73)	<0.001
Pruritus NRS	6.6 ± 1.0	4.8 ± 1.2	−1.8 ± 0.5	6.8 ± 1.1	4.1 ± 1.3	−2.7 ± 0.6	−0.94 (−1.15, −0.72)	<0.001
DLQI	12.2 ± 3.5	7.4 ± 3.5	−4.7 ± 1.7	13.0 ± 3.3	5.2 ± 3.1	−7.7 ± 1.8	−2.86 (−3.47, −2.26)	<0.001

Data are presented as mean ± SD. Baseline values are summarized in the randomized population; week-12 and change values are summarized among participants with week-12 assessment. Adjusted mean differences at week 12 were estimated using ANCOVA with the week-12 score as the dependent variable, treatment group as the main factor, and baseline score as a covariate.

SCORAD showed a similar pattern. Mean SCORAD changed from 41.1 ± 8.2 to 24.1 ± 9.4 in the placebo group and from 42.7 ± 8.6 to 15.9 ± 8.7 in the Qing-Re-Qu-Shi group, corresponding to mean changes of −16.6 ± 4.8 and −26.5 ± 5.3. The adjusted mean difference at week 12 was −9.73 (95% CI − 11.54 to −7.93; *p* < 0.001).

The same direction was seen for other symptom-related outcomes. Compared with placebo, the Qing-Re-Qu-Shi group showed larger improvements in POEM (adjusted mean difference −3.39, 95% CI − 4.06 to −2.73; *p* < 0.001), pruritus NRS (−0.94, 95% CI − 1.15 to −0.72; *p* < 0.001), and DLQI (−2.86, 95% CI − 3.47 to −2.26; *p* < 0.001).

### 3.4 Responder outcomes

Responder analyses in the intention-to-treat population also favored Qing-Re-Qu-Shi (Table 3). EASI-50 was achieved by 49 of 76 participants (64.5%) in the Qing-Re-Qu-Shi group, compared with 12 of 76 (15.8%) in the placebo group, corresponding to a risk ratio of 4.08 (95% CI 2.37 to 7.04; *p* < 0.001). EASI-75 was reached by 15 of 76 participants (19.7%) in the Qing-Re-Qu-Shi group and 2 of 76 (2.6%) in the placebo group, with a risk ratio of 7.50 (95% CI 1.78 to 31.68; *p* = 0.001). Absolute EASI improvement thresholds supported the clinical meaningfulness of the treatment effect: improvement of at least 4 points was achieved by 62 of 76 participants (81.6%) in the Qing-Re-Qu-Shi group and 47 of 76 (61.8%) in the placebo group, and improvement of at least 6.6 points by 55 of 76 (72.4%) and 14 of 76 (18.4%), respectively (Supplementary Table S8).

**Table 3:** Responder outcomes at week 12 in the intention-to-treat population.

Endpoint	Placebo	Qing-Re-Qu-Shi formula	Risk ratio (95% CI)	*p* value
EASI-50 response	12/76 (15.8%)	49/76 (64.5%)	4.08 (2.37, 7.04)	<0.001
EASI-75 response	2/76 (2.6%)	15/76 (19.7%)	7.50 (1.78, 31.68)	0.001

Data are presented as n/N (%). Responder analyses were performed according to the intention-to-treat principle; participants without week-12 assessment were counted as non-responders. *p* values were calculated using Fisher’s exact test.

### 3.5 Gut microbiota diversity

**Figure 3:**
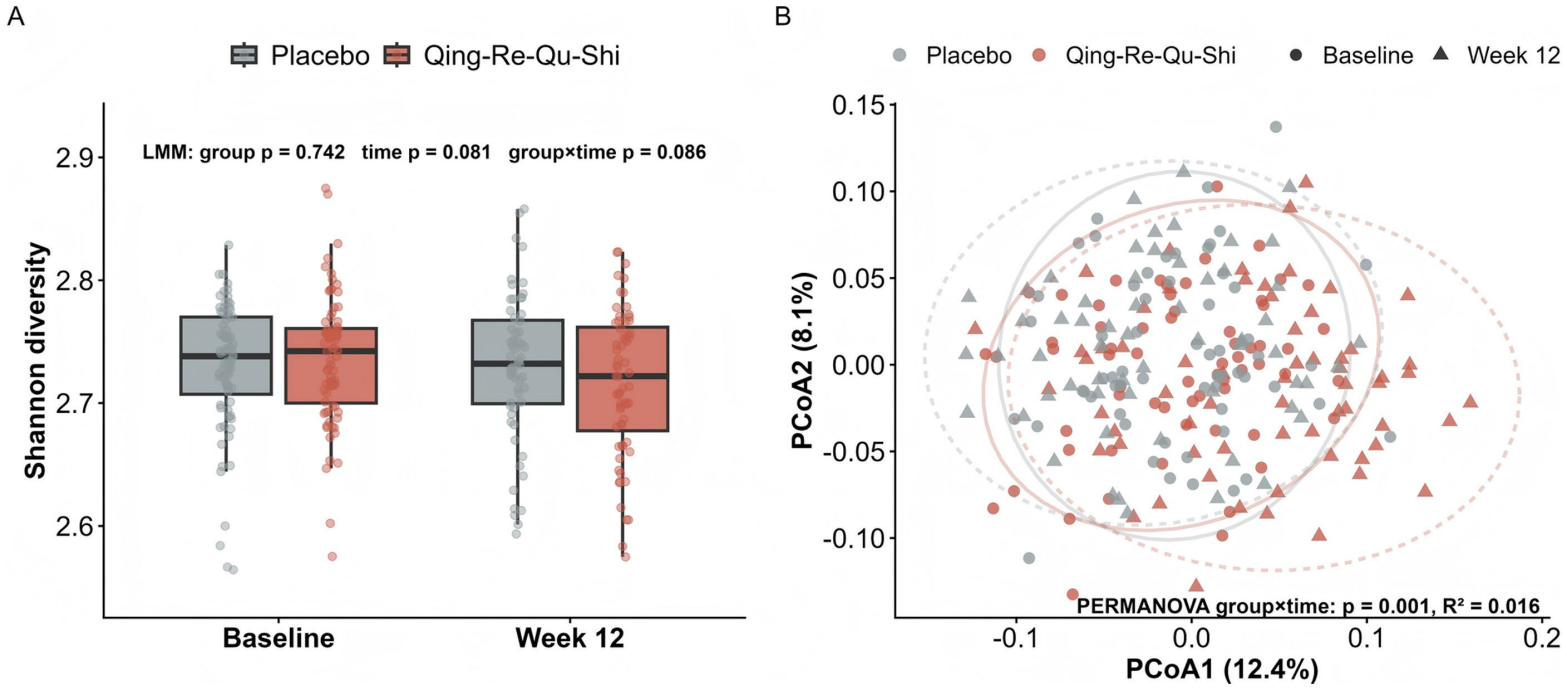
Alpha and *β* diversity of the gut microbiota across treatment groups and time points. **(A)** Shannon diversity according to treatment group and time point. Boxplots show the distribution of Shannon diversity with overlaid individual observations. *p* values were derived from linear mixed-effects models including group, time point, and their interaction, with participant-level random intercepts. **(B)** Principal coordinates analysis (PCoA) based on Bray-Curtis distances for baseline and week-12 samples. PERMANOVA used permutations restricted within participant ID and showed a statistically significant but small group × time effect.

Changes in overall alpha diversity were limited (Figure 3A). Shannon diversity remained similar across groups and time points. In the linear mixed-effects model, there was no clear evidence of a group effect (*p* = 0.742), time effect (*p* = 0.081), or group × time interaction (*p* = 0.086; Supplementary Table S14). Read-depth and feature-filtering summaries are provided in Supplementary Tables S12, S13.

By contrast, *β* diversity analysis suggested a modest but statistically detectable shift in overall microbial community structure (Figure 3B). PCoA based on Bray-Curtis distances showed substantial overlap between groups and time points, but repeated-measures-aware PERMANOVA identified a significant group × time effect (*p* = 0.001), with a small effect size (*R*^2^ = 0.016; Supplementary Table S15). PERMDISP did not indicate a significant difference in dispersion across group-time strata (*p* = 0.085; Supplementary Table S16), and the adjusted between-group difference in within-subject Bray-Curtis distance from baseline to week 12 was small (0.002; 95% CI −0.007 to 0.011; *p* = 0.641; Supplementary Tables S17, S18). Taken together, these findings suggest that the intervention was associated more with targeted compositional remodeling of specific features than with a large community-level shift.

### 3.6 Differential microbial taxa

**Figure 4:**
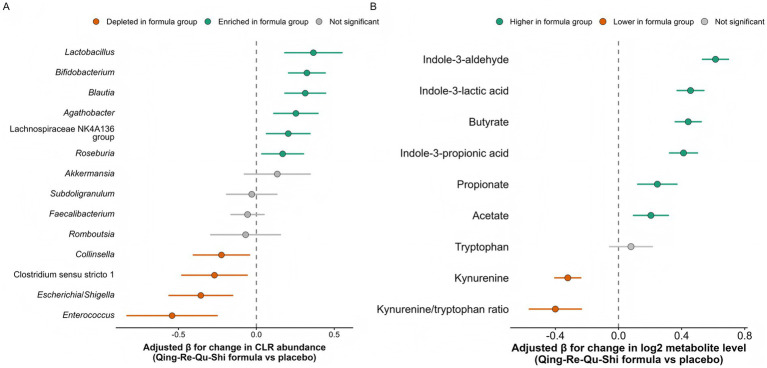
Differential changes in gut microbial taxa and targeted serum metabolites between treatment groups. **(A)** Adjusted between-group differences in change from baseline to week 12 for tested gut microbial taxa, expressed as *β* coefficients for CLR-transformed abundance. **(B)** Adjusted between-group differences in change from baseline to week 12 for targeted serum metabolites, expressed as *β* coefficients for log2-transformed metabolite levels. Points indicate adjusted *β* estimates and horizontal lines indicate 95% confidence intervals. Models were adjusted for baseline feature level, age, and sex. Gray indicates non-significant features after multiple-testing correction.

At the taxon level, full-feature CLR analyses showed several FDR-significant between-group differences in change from baseline to week 12 (Figure 4A, Table 4, Supplementary Table S19, and Supplementary Figure S1). The strongest positive changes were observed for Blautia (adjusted *β* 0.328, 95% CI 0.216 to 0.441; FDR-adjusted *p* < 0.001), Bifidobacterium (0.319, 95% CI 0.207 to 0.431; FDR-adjusted *p* < 0.001), Lactobacillus (0.350, 95% CI 0.189 to 0.511; FDR-adjusted *p* < 0.001), and Agathobacter (0.206, 95% CI 0.078 to 0.334; FDR-adjusted *p* = 0.006).

**Table 4:** FDR-significant differential changes in gut microbial taxa in the full-feature CLR analysis.

Taxon	Adjusted *β* for change (95% CI)	*p* value	FDR-adjusted *p*
Blautia	0.328 (0.216, 0.441)	<0.001	<0.001
Bifidobacterium	0.319 (0.207, 0.431)	<0.001	<0.001
Lactobacillus	0.350 (0.189, 0.511)	<0.001	<0.001
Agathobacter	0.206 (0.078, 0.334)	0.002	0.006
Escherichia/Shigella	−0.350 (−0.524, −0.176)	<0.001	<0.001
Enterococcus	−0.512 (−0.788, −0.235)	<0.001	0.001
Prevotella	−0.122 (−0.218, −0.026)	0.013	0.037
Collinsella	−0.202 (−0.370, −0.035)	0.018	0.045

Adjusted *β* values represent between-group differences in change from baseline to week 12 in CLR-transformed abundance. Linear models were adjusted for baseline abundance, age, and sex. False discovery rate was controlled using the Benjamini-Hochberg procedure. Full results for all tested taxa are provided in Supplementary Table S19 and Supplementary Figure S1.

At the same time, Escherichia/Shigella (adjusted *β* − 0.350, 95% CI −0.524 to −0.176; FDR-adjusted *p* < 0.001), Enterococcus (−0.512, 95% CI −0.788 to −0.235; FDR-adjusted *p* = 0.001), Prevotella (−0.122, 95% CI −0.218 to −0.026; FDR-adjusted *p* = 0.037), and Collinsella (−0.202, 95% CI −0.370 to −0.035; FDR-adjusted *p* = 0.045) were lower in the Qing-Re-Qu-Shi group. Roseburia, Lachnospiraceae NK4A136 group, and Clostridium sensu stricto 1 showed directionally consistent changes but did not remain statistically significant after full-feature FDR correction.

**Figure 5:**
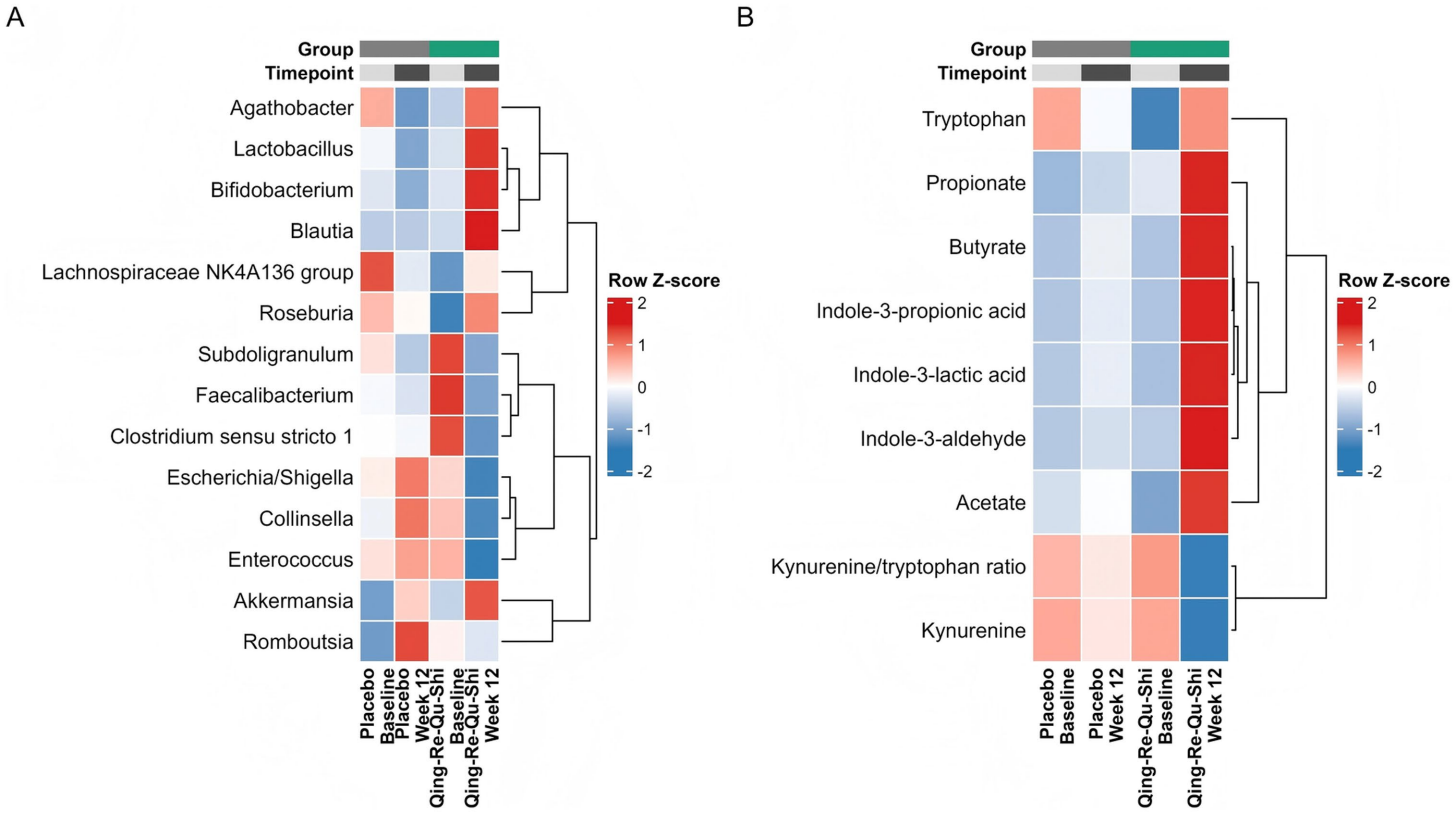
Heatmaps of row-scaled mean microbial and serum metabolite profiles across treatment groups and time points. **(A)** Row-scaled mean relative abundance of selected gut microbial taxa. **(B)** Row-scaled mean log2-transformed levels of targeted serum metabolites. Values represent row-wise *Z*-scores calculated within each feature. Columns are ordered as placebo baseline, placebo week 12, Qing-Re-Qu-Shi baseline, and Qing-Re-Qu-Shi week 12.

The row-scaled heatmap showed the same overall pattern, with treatment-associated enriched taxa tending to be relatively higher in the Qing-Re-Qu-Shi week-12 samples and reduced taxa tending to be relatively lower (Figure 5A).

### 3.7 Targeted metabolites

Targeted serum metabolomics revealed coordinated changes in circulating microbiota-related metabolites that paralleled the taxonomic findings (Figure 4B, Table 5, Supplementary Table S20, and Supplementary Figure S2). Qing-Re-Qu-Shi was associated with higher short-chain fatty acids, including butyrate (adjusted *β* 0.368, 95% CI 0.301 to 0.434; FDR-adjusted *p* < 0.001), propionate (0.225, 95% CI 0.131 to 0.318; FDR-adjusted *p* < 0.001), and acetate (0.160, 95% CI 0.077 to 0.243; FDR-adjusted *p* < 0.001).

**Table 5:** Differential changes in targeted serum metabolites between treatment groups.

Pathway	Metabolite	Adjusted *β* for change (95% CI)	*p* value	FDR-adjusted *p*
Short-chain fatty acids	Butyrate	0.368 (0.301, 0.434)	<0.001	<0.001
Short-chain fatty acids	Propionate	0.225 (0.131, 0.318)	<0.001	<0.001
Short-chain fatty acids	Acetate	0.160 (0.077, 0.243)	<0.001	<0.001
Indole metabolites	Indole-3-aldehyde	0.507 (0.439, 0.575)	<0.001	<0.001
Indole metabolites	Indole-3-lactic acid	0.369 (0.300, 0.437)	<0.001	<0.001
Indole metabolites	Indole-3-propionic acid	0.336 (0.265, 0.408)	<0.001	<0.001
Tryptophan/kynurenine axis	Tryptophan	0.036 (−0.062, 0.134)	0.470	0.470
Tryptophan/kynurenine axis	Kynurenine	−0.260 (−0.329, −0.192)	<0.001	<0.001
Tryptophan/kynurenine axis	Kynurenine/tryptophan ratio	−0.307 (−0.436, −0.178)	<0.001	<0.001

Adjusted *β* values represent between-group differences in change from baseline to week 12 in log2-transformed metabolite levels. Linear models were adjusted for baseline metabolite level, age, and sex. False discovery rate was controlled using the Benjamini-Hochberg procedure. Full targeted serum metabolite results are provided in Supplementary Table S20 and Supplementary Figure S2.

Indole-related metabolites also increased. The strongest signal was seen for indole-3-aldehyde (adjusted β 0.507, 95% CI 0.439 to 0.575; FDR-adjusted *p* < 0.001), followed by indole-3-lactic acid (0.369, 95% CI 0.300 to 0.437; FDR-adjusted *p* < 0.001) and indole-3-propionic acid (0.336, 95% CI 0.265 to 0.408; FDR-adjusted *p* < 0.001). In contrast, kynurenine decreased (adjusted *β* − 0.260, 95% CI −0.329 to −0.192; FDR-adjusted *p* < 0.001), and the kynurenine/tryptophan ratio also declined (−0.307, 95% CI −0.436 to −0.178; FDR-adjusted *p* < 0.001). Tryptophan itself did not show a significant between-group difference (0.036, 95% CI −0.062 to 0.134; *p* = 0.470; FDR-adjusted *p* = 0.470).

The heatmap of row-scaled mean levels supported the same picture: SCFA- and indole-related metabolites were relatively higher at week 12 in the Qing-Re-Qu-Shi group, whereas kynurenine-related measures showed the opposite pattern (Figure 5B).

### 3.8 Integrated associations between omics changes and clinical improvement

**Figure 6:**
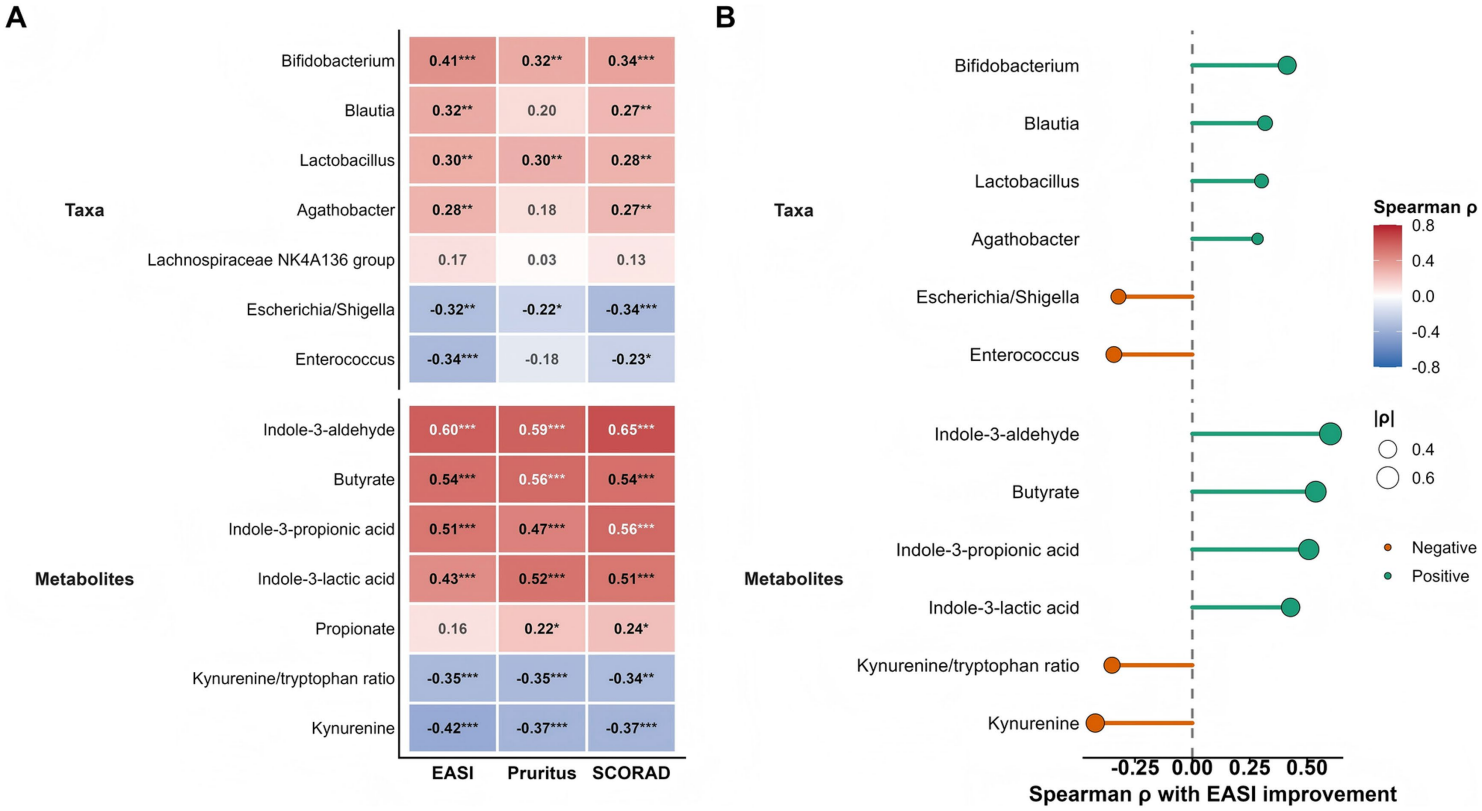
Integrated associations of fecal microbial and serum metabolic features with clinical improvement. **(A)** Heatmap of Spearman correlations between changes in selected taxa/serum metabolites and improvement in EASI, pruritus, and SCORAD among participants with paired omics data and observed week-12 outcomes. Values in the cells denote Spearman’s *ρ*; significance symbols indicate false discovery rate-adjusted thresholds. **(B)** Lollipop plot showing the direction and magnitude of the association between selected features and EASI improvement. Point size is proportional to the absolute Spearman ρ.

Cross-domain correlation analyses were performed among 102 participants who had paired omics data and observed week-12 clinical outcome data. These analyses showed that the microbial and serum-metabolite shifts were directionally aligned with clinical improvement (Figure 6A and Supplementary Table S23). Among metabolites, indole-3-aldehyde showed the strongest positive correlations with improvement in EASI (*ρ* = 0.60), pruritus (*ρ* = 0.59), and SCORAD (*ρ* = 0.65). Butyrate was also consistently correlated with symptom improvement (*ρ* = 0.54, 0.56, and 0.54, respectively). Indole-3-propionic acid and indole-3-lactic acid showed the same pattern, with moderate positive correlations across all three clinical measures.

Among taxa, Bifidobacterium, Blautia, Lactobacillus, and Agathobacter were positively correlated with improvement, whereas Escherichia/Shigella and Enterococcus were negatively correlated. Kynurenine and the kynurenine/tryptophan ratio were inversely correlated with clinical improvement across the three symptom domains. Correlations between treatment-associated genera and indole-related metabolites were also observed, but these results should be interpreted as cross-domain concordance rather than proof that the enriched genera directly produced the measured metabolites (Supplementary Table S22).

The EASI-focused lollipop plot highlighted the same rank order more clearly (Figure 6B). The strongest positive correlates of EASI improvement were indole-3-aldehyde, butyrate, indole-3-propionic acid, indole-3-lactic acid, and Bifidobacterium, while kynurenine, the kynurenine/tryptophan ratio, Escherichia/Shigella, and Enterococcus were among the strongest negative correlates. Within the Qing-Re-Qu-Shi arm, exploratory comparisons of omics changes between EASI-50/EASI-75 responders and non-responders did not identify robust differences after FDR correction (Supplementary Table S21).

### 3.9 Safety and completion

Safety findings were reassuring overall (Supplementary Table S1). Any gastrointestinal adverse event occurred in 12 of 76 participants (15.8%) in the Qing-Re-Qu-Shi group and 6 of 76 (7.9%) in the placebo group (Fisher exact *p* = 0.209). Other mild adverse events were reported in 15 of 76 participants (19.7%) in the Qing-Re-Qu-Shi group and 18 of 76 (23.7%) in the placebo group (*p* = 0.694). Any recorded adverse event occurred in 24 of 76 participants (31.6%) in the Qing-Re-Qu-Shi group and 22 of 76 (28.9%) in the placebo group (*p* = 0.860). No participant withdrew because of an adverse event or safety reason. Adherence was similar between groups, and per-protocol and adherence-adjusted sensitivity analyses were consistent with the primary results (Supplementary Tables S9, S10). Completion of week-12 follow-up was numerically higher in the Qing-Re-Qu-Shi group (64/76, 84.2%) than in the placebo group (60/76, 78.9%).

## 4 Discussion

In this randomized, placebo-controlled trial, Qing-Re-Qu-Shi formula produced consistent clinical benefit across multiple symptom domains. Compared with placebo, the active-treatment group showed larger improvements in EASI, SCORAD, POEM, pruritus NRS, and DLQI, together with substantially higher EASI-50 and EASI-75 response rates. These efficacy findings were accompanied by directional shifts in gut microbial composition and targeted circulating microbiota-related metabolites, whereas changes in global community diversity were modest. Taken together, the data suggest that the benefit of the intervention was associated more with selective ecological and metabolic remodeling than with a broad increase in overall gut microbial diversity.

This pattern is biologically plausible in the context of current understanding of atopic dermatitis. AD is now viewed as a disease driven by the interaction of epidermal barrier dysfunction, immune dysregulation, and microbial imbalance, rather than by a single dominant pathway (4, 24). In that framework, an intervention does not need to normalize the entire microbiome to be clinically relevant. A more realistic scenario is the selective enrichment or depletion of specific taxa and metabolites that are aligned with epithelial homeostasis, immune restraint, or inflammatory activation. That is essentially the pattern we observed, although the data remain associative.

One of the more interesting aspects of our data is the disconnect between diversity metrics and feature-level results. Shannon diversity did not change materially, and the PERMANOVA effect size for *β* diversity was statistically significant but small. PERMDISP and within-subject Bray-Curtis analyses further suggested that the global community-level shift was limited. This is not necessarily a weakness. Adult AD studies have often reported heterogeneous or limited diversity differences, even when compositional differences in specific organisms are present (6). In addition, earlier work has linked eczema severity more closely to the abundance of particular butyrate-associated or dysbiosis-related taxa than to diversity alone (7, 8). Our findings fit that literature reasonably well: the signal in our dataset was carried by selected feature-level taxa and metabolites, not by a dramatic shift in alpha diversity or by a microbiome-wide reset.

At the taxonomic level, Qing-Re-Qu-Shi was associated with increased Blautia, Bifidobacterium, Lactobacillus, and Agathobacter, together with reduced Escherichia/Shigella, Enterococcus, Prevotella, and Collinsella after full-feature FDR correction. Roseburia, Lachnospiraceae NK4A136 group, and Clostridium sensu stricto 1 showed directionally consistent but FDR-non-significant changes. We would be cautious about labeling any genus as universally beneficial or deleterious, because genus-level signals can conceal considerable species- and strain-level functional heterogeneity. Even so, the overall direction is coherent. Adult AD literature has repeatedly pointed toward enrichment of potentially dysbiosis-associated taxa such as Enterobacteriaceae-related organisms and reduction in short-chain-fatty-acid-related ecological functions (6, 7). Likewise, adult and mixed-age probiotic or microbiota-directed studies have suggested that strains containing Lactobacillus and/or Bifidobacterium may be associated with symptom improvement, although the evidence remains strain-specific and not uniformly positive across all trials (12–14, 25). Our results do not prove that these taxa are the mediators of treatment response, but they do place the observed taxonomic shifts within an already recognizable biological pattern.

The metabolite findings strengthen that interpretation. Qing-Re-Qu-Shi increased butyrate, propionate, acetate, indole-3-aldehyde, indole-3-lactic acid, and indole-3-propionic acid, while reducing kynurenine and the kynurenine/tryptophan ratio. Among these, the rise in butyrate and indole derivatives is particularly noteworthy. Prior human and experimental work has linked AD to lower fecal SCFA output or reduced abundance of SCFA-producing ecological functions (7, 8, 26). Experimental models have further shown that butyrate or acetate augmentation can attenuate AD-like inflammation (9, 27). In parallel, microbial tryptophan metabolites such as indole derivatives can activate the aryl hydrocarbon receptor (AhR), a pathway increasingly recognized as relevant to skin barrier function and cutaneous immune regulation (11, 28). The positive correlations between treatment-associated genera and indole metabolites are compatible with this biology but do not establish which organisms produced these metabolites. Our dataset did not test AhR signaling directly, but the increase in indole-3-aldehyde and related metabolites is directionally compatible with that axis.

The tryptophan–kynurenine findings deserve a more careful interpretation. We observed lower kynurenine and a lower kynurenine/tryptophan ratio in the Qing-Re-Qu-Shi group, whereas tryptophan itself was not significantly altered. This is an internally coherent result and may reflect a shift away from inflammatory tryptophan catabolism rather than simply an increase in substrate availability. Prior studies have reported altered kynurenine-pathway activity in allergic disease, including AD, and inflammatory skin tissue can show increased expression of tryptophan-catabolic enzymes such as kynureninase (29, 30). That said, the clinical meaning of lower circulating or fecal kynurenine-related indices in AD is not fully settled, and our results should not be overread as proving normalization of a single pathway. A more restrained interpretation is that Qing-Re-Qu-Shi was associated with a metabolite profile that favored SCFA- and indole-related signals while reducing kynurenine-related inflammatory signatures.

The integrated correlation analyses help connect these omics findings to the clinical data, but they also set a boundary on what can be claimed. Features that increased with treatment and are biologically plausible in AD-related gut-skin signaling, such as Bifidobacterium, Lactobacillus, butyrate, indole-3-aldehyde, and indole-3-propionic acid, were positively associated with improvement in EASI, pruritus, and SCORAD. Conversely, Enterococcus, Escherichia/Shigella, kynurenine, and the kynurenine/tryptophan ratio were inversely associated with clinical improvement. This cross-domain concordance is encouraging, because it argues against the microbiome and serum-metabolite findings being merely incidental. Still, these are correlations, not mediation results. Exploratory mediation analyses were unstable because the complete-case sample for joint treatment-mediator-outcome modeling was limited and indirect-effect confidence intervals crossed zero (Supplementary Table S24). The observed omics changes may therefore represent causal contributors to clinical improvement, secondary consequences of reduced inflammation or scratching, or parallel effects of the intervention on host and microbial physiology.

Another point worth noting is that the clinical benefit appeared larger and cleaner than the diversity-level microbiome signal. In practice, that is not surprising. Clinical outcomes in AD can improve through several partially overlapping routes: reduced inflammation, better barrier function, less itch-related scratching, changes in circulating microbiota-related metabolites, or combinations of these. A gut-directed effect may therefore be clinically meaningful even when the community-level microbiome shift is visually modest. Indeed, prior probiotic studies in adults have also reported clinical improvement without a simple one-to-one correspondence with broad microbiome readouts (12–14, 25). Our data fit that more nuanced model better than an all-or-none microbiome-reset hypothesis. A larger longitudinal study with more frequent stool and clinical sampling, shotgun metagenomics, strain-level profiling, and prespecified causal-mediation modeling would be needed to test mediation more directly.

The safety profile in our study was also reassuring. Gastrointestinal adverse events were numerically more common in the Qing-Re-Qu-Shi group, but the difference was not statistically significant, and other mild adverse events were similar between groups. No strong safety signal emerged over 12 weeks. That matters, because any microbiota-directed adjunct for AD needs to offer not only biological plausibility but also tolerability and practical feasibility.

This study also has limitations. First, although the trial was randomized, the omics analyses were performed in participants with paired fecal 16S and serum metabolomics data rather than in the full randomized population, and cross-domain association analyses were limited to 102 participants with paired omics data and observed week-12 clinical outcomes. Second, the microbiome analysis was based on 16S rRNA profiling, which limits taxonomic resolution and does not directly measure strain-specific function. Third, the serum metabolomics panel was targeted rather than untargeted; that improved interpretability, but it necessarily restricted the range of detectable pathways. Fourth, the integrated analyses were correlational, and exploratory mediation analyses did not yield stable indirect effects suitable for formal interpretation. Fifth, rescue topical therapy was standardized but not quantitatively captured (Supplementary Table S11), adverse-event severity and relatedness were not recorded in sufficient granularity for detailed safety grading, and dietary habits were not systematically measured, which is relevant for microbiome and serum-metabolite interpretation. Finally, the trial was not prospectively registered and was conducted in a single regional adult population with moderate AD; the generalizability of the findings to mild or severe AD, pediatric patients, patients receiving biologics or JAK inhibitors, and populations outside the study region remains uncertain.

Despite these caveats, the overall picture is coherent. Qing-Re-Qu-Shi improved the major clinical manifestations of moderate AD, while shifting the gut ecosystem and circulating metabolite profile toward higher SCFA and indole-related signals, lower kynurenine-related signals, enrichment of several treatment-associated genera, and depletion of selected dysbiosis-associated genera. The diversity findings were modest, but the feature-level and cross-domain findings were directionally consistent. On balance, these results support the view that microbiome-linked circulating metabolic remodeling may be one plausible biological companion of clinical response, while stopping short of claiming that it is the sole mechanism of benefit.

## Data Availability

The de-identified processed clinical data, processed 16S rRNA microbiome tables, targeted serum metabolomics matrices, derived statistical result tables, and figure source data supporting the findings of this study are deposited in the Zenodo repository, DOI: 10.5281/zenodo.20801058.
