# Supplementary material for: Qing-Re-Qu-Shi formula improves clinical outcomes and is accompanied by gut microbiota and microbial metabolite remodeling in adults with moderate atopic dermatitis: a randomized placebo-controlled multi-omics trial
Source: Front Med (Lausanne). 2026 Jul 31;13:1847003. doi: 10.3389/fmed.2026.1847003 (PMC13475060; doi:10.3389/fmed.2026.1847003)
Supplement: Supplementary file 1 [file Table_1.docx]

**Supplementary Material**

Qing-Re-Qu-Shi Formula Improves Clinical Outcomes and Is Accompanied by Gut Microbiota and Microbial Metabolite Remodeling in Adults With Moderate Atopic Dermatitis: A Randomized Placebo-Controlled Multi-Omics Trial

**Supplementary Methods**

**Study drug and placebo.** Qing-Re-Qu-Shi formula was administered as a fixed granule preparation at one 10-g sachet twice daily for 12 weeks. The formulation was designed according to the traditional therapeutic principle of clearing heat, removing dampness, cooling blood, and relieving pruritus. The placebo consisted of inert granules based on dextrin and starch excipients, with food-grade colorant and flavoring agents to mimic the appearance, smell, taste, packaging, and administration schedule of the active granules.

**16S rRNA sequencing. The V3-V4 region of the 16S rRNA gene was amplified from fecal DNA using dual-index primers 338F (5'-ACTCCTACGGGAGGCAGCAG-3') and 806R (5'-GGACTACHVGGGTWTCTAAT-3'). PCR reactions were performed using 5× FastPfu DNA Polymerase, and high-quality amplicons were sequenced on the Illumina NovaSeq PE250 platform. Raw reads were processed in QIIME 2 version 2021.8 using DADA2 to generate amplicon sequence variants. Taxonomy was assigned using a Naive Bayes classifier trained on the SILVA 138 99% reference database. Features present in only a single sample were filtered out, and genus-level features with mean relative abundance below 0.01% or prevalence below 25% were excluded from downstream feature testing. Zero values were handled using a pseudocount before centered log-ratio transformation.**

**Targeted serum metabolomics. Targeted profiling of circulating microbiota-related metabolites was conducted using serum specimens. Frozen serum samples were thawed on ice and extracted with acetonitrile:methanol (1:4, v/v) containing isotope-labeled internal standards. Serum extracts were analyzed using LC-ESI-MS/MS on an ExionLC AD UPLC system coupled to a QTRAP 6500+ mass spectrometer (AB Sciex). Extracts were separated on a Waters HSS T3 column (1.8 µm, 2.1 mm × 100 mm) using water and acetonitrile containing 0.1% formic acid as mobile phases. Scheduled multiple reaction monitoring was used for quantification based on predefined precursor-product ion pairs and retention-time information. Pooled quality-control samples were injected after every ten analytical samples. Metabolites with detection rates below 80% or QC CV above 50% were excluded, missing values were imputed using K-nearest neighbors, and batch effects were corrected using support vector regression. Targeted serum metabolites included short-chain fatty acids, tryptophan, kynurenine, the kynurenine/tryptophan ratio, and indole-related metabolites.**

**Supplementary statistical analyses. Additional analyses included baseline-imbalance-adjusted ANCOVA, missing outcome sensitivity analyses, adherence and per-protocol sensitivity analyses, microbiome QC, repeated-measures-aware PERMANOVA with PERMDISP, within-subject Bray-Curtis distance analysis, full-feature CLR-based taxa testing, full targeted serum-metabolite testing, QRQS-arm responder/non-responder omics comparisons, taxa-indole serum-metabolite correlations, cross-domain Spearman correlations, and exploratory mediation diagnostics.**

**Table S1. Expanded safety outcomes and study completion by treatment group.**

| **Endpoint** | **Placebo** | **Qing-Re-Qu-Shi** | **Risk ratio (95% CI)** | **P value** |
| --- | --- | --- | --- | --- |
| Any gastrointestinal adverse event | 6/76 (7.9%) | 12/76 (15.8%) | 2.00 (0.79, 5.05) | 0.209 |
| Any other mild adverse event | 18/76 (23.7%) | 15/76 (19.7%) | 0.83 (0.45, 1.53) | 0.694 |
| Any recorded adverse event | 22/76 (28.9%) | 24/76 (31.6%) | 1.09 (0.67, 1.77) | 0.86 |
| Withdrawal due to adverse event or safety reason | 0/76 (0.0%) | 0/76 (0.0%) | 1.00 (0.02, 49.76) | 1.0 |

*Data are presented as n/N (%). P values were calculated using Fisher exact tests. Severity and relatedness grading were not available in the analysis dataset.*

**Table S2. Qing-Re-Qu-Shi formula composition used for manuscript reporting.**

| **Herb (Latin name)** | **Pinyin/common Chinese name** | **Traditional role** | **Raw-herb equivalent amount** |
| --- | --- | --- | --- |
| Sophora flavescens Aiton | Ku Shen | Clear heat and dry dampness | 10 g |
| Phellodendron chinense C.K.Schneid. | Huang Bai | Clear damp-heat | 10 g |
| Smilax glabra Roxb. | Tu Fu Ling | Remove dampness and detoxify | 15 g |
| Coix lacryma-jobi L. var. ma-yuen | Yi Yi Ren | Strengthen spleen and drain dampness | 15 g |
| Dictamnus dasycarpus Turcz. | Bai Xian Pi | Clear damp-heat and relieve pruritus | 10 g |
| Kochia scoparia (L.) Schrad. | Di Fu Zi | Remove dampness and relieve pruritus | 10 g |
| Lonicera japonica Thunb. | Jin Yin Hua | Clear heat and detoxify | 10 g |
| Forsythia suspensa (Thunb.) Vahl | Lian Qiao | Clear heat and disperse toxin | 10 g |
| Rehmannia glutinosa (Gaertn.) DC. | Sheng Di Huang | Cool blood and nourish yin | 12 g |
| Paeonia lactiflora Pall. | Chi Shao | Cool blood and activate blood | 10 g |
| Glycyrrhiza uralensis Fisch. ex DC. | Gan Cao | Harmonize formula and detoxify | 6 g |

*Raw-herb equivalent amounts are shown according to the study-drug manufacturing record used for manuscript reporting.*

**Table S3. Analysis populations and cross-domain overlap.**

| **Analysis population** | **Placebo** | **Qing-Re-Qu-Shi** | **Total** |
| --- | --- | --- | --- |
| Randomized participants | 76 | 76 | 152 |
| Completed week-12 clinical follow-up | 60 | 64 | 124 |
| Participants with paired fecal 16S data and paired serum metabolomics data | 60 | 64 | 124 |
| Participants with paired fecal 16S data, paired serum metabolomics data, and week-12 clinical outcome data for cross-domain association analyses | 48 | 54 | 102 |

*The clinical-completion set and paired-omics set were not completely identical; 102 participants had paired fecal 16S data, paired serum metabolomics data, and observed week-12 clinical outcomes for cross-domain analyses.*

**Table S4. Baseline standardized mean differences for all baseline variables.**

| **Variable** | **abs_smd** | **interpretation** |
| --- | --- | --- |
| family_history_atopy | 0.2809757434745082 | Moderate or greater |
| disease_duration_years | 0.269179231654461 | Moderate or greater |
| asthma | 0.2574596813804536 | Moderate or greater |
| dlqi_baseline | 0.2395389823518068 | Small |
| total_ig_e_baseline_iu_m_l | 0.2060542751786243 | Small |
| pruritus_nrs_baseline | 0.2049386389400353 | Small |
| scorad_baseline | 0.1893618925932015 | Small |
| baseline_iga | 0.1818749744238325 | Small |
| tewl_baseline_g_m2_h | 0.163975611812662 | Small |
| bmi_kg_m2 | 0.1433741935540573 | Small |
| tarc_baseline_pg_m_l | 0.128300562595872 | Small |
| poem_baseline | 0.0985113614212062 | Negligible |
| easi_baseline | 0.0984213013690944 | Negligible |
| age_years | 0.0750411392450159 | Negligible |
| eosinophils_baseline_10e9_l | 0.0609190466142071 | Negligible |
| sex | 0.0526680653149723 | Negligible |
| allergic_rhinitis | 0.0262559755554208 | Negligible |

*SMD values are absolute standardized mean differences. Variables with SMD ≥0.25 were considered modestly imbalanced.*

**Table S5. Baseline-imbalance-adjusted sensitivity analyses.**

| **Endpoint** | **Model** | **Additional covariates** | **n** | **Adjusted mean difference (95% CI)** | **P value** |
| --- | --- | --- | --- | --- | --- |
| EASI | Complete-case ANCOVA | family_history_atopy, disease_duration_years, asthma | 124 | -3.78 (-4.54, -3.01) | <0.001 |
| EASI | ANCOVA additionally adjusted for baseline imbalances | family_history_atopy, disease_duration_years, asthma | 124 | -3.79 (-4.59, -2.99) | <0.001 |
| SCORAD | Complete-case ANCOVA | family_history_atopy, disease_duration_years, asthma | 124 | -9.73 (-11.54, -7.93) | <0.001 |
| SCORAD | ANCOVA additionally adjusted for baseline imbalances | family_history_atopy, disease_duration_years, asthma | 124 | -9.89 (-11.77, -8.02) | <0.001 |
| POEM | Complete-case ANCOVA | family_history_atopy, disease_duration_years, asthma | 124 | -3.39 (-4.06, -2.73) | <0.001 |
| POEM | ANCOVA additionally adjusted for baseline imbalances | family_history_atopy, disease_duration_years, asthma | 124 | -3.53 (-4.22, -2.85) | <0.001 |
| Pruritus NRS | Complete-case ANCOVA | family_history_atopy, disease_duration_years, asthma | 124 | -0.94 (-1.15, -0.72) | <0.001 |
| Pruritus NRS | ANCOVA additionally adjusted for baseline imbalances | family_history_atopy, disease_duration_years, asthma | 124 | -0.98 (-1.21, -0.76) | <0.001 |
| DLQI | Complete-case ANCOVA | family_history_atopy, disease_duration_years, asthma | 124 | -2.86 (-3.47, -2.26) | <0.001 |
| DLQI | ANCOVA additionally adjusted for baseline imbalances | family_history_atopy, disease_duration_years, asthma | 124 | -2.96 (-3.59, -2.34) | <0.001 |

*Models additionally adjusted for family history of atopy, disease duration, and asthma.*

**Table S6. Missing week-12 continuous outcome sensitivity analyses.**

| **Endpoint** | **Model** | **n** | **Treatment effect (95% CI)** | **P value** |
| --- | --- | --- | --- | --- |
| EASI | Complete-case ANCOVA | 124 | -3.78 (-4.54, -3.01) | <0.001 |
| EASI | MMRM-style LMM using all available visits | 152 | -3.50 (-4.79, -2.21) | <0.001 |
| EASI | LOCF sensitivity ANCOVA | 152 | -3.74 (-4.53, -2.96) | <0.001 |
| EASI | BOCF sensitivity ANCOVA | 152 | -3.47 (-4.57, -2.36) | <0.001 |
| EASI | Multiple imputation ANCOVA, m=50 | 152 | -3.81 (-4.59, -3.03) | <0.001 |
| SCORAD | Complete-case ANCOVA | 124 | -9.73 (-11.54, -7.93) | <0.001 |
| SCORAD | MMRM-style LMM using all available visits | 152 | -8.39 (-11.23, -5.55) | <0.001 |
| SCORAD | LOCF sensitivity ANCOVA | 152 | -9.90 (-11.92, -7.88) | <0.001 |
| SCORAD | BOCF sensitivity ANCOVA | 152 | -9.18 (-12.28, -6.09) | <0.001 |
| SCORAD | Multiple imputation ANCOVA, m=50 | 152 | -9.66 (-11.31, -8.00) | <0.001 |
| POEM | Complete-case ANCOVA | 124 | -3.39 (-4.06, -2.73) | <0.001 |
| POEM | MMRM-style LMM using all available visits | 152 | -3.11 (-4.29, -1.93) | <0.001 |
| POEM | LOCF sensitivity ANCOVA | 152 | -3.48 (-4.19, -2.76) | <0.001 |
| POEM | BOCF sensitivity ANCOVA | 152 | -3.16 (-4.20, -2.11) | <0.001 |
| POEM | Multiple imputation ANCOVA, m=50 | 152 | -3.52 (-4.14, -2.89) | <0.001 |
| Pruritus NRS | Complete-case ANCOVA | 124 | -0.94 (-1.15, -0.72) | <0.001 |
| Pruritus NRS | MMRM-style LMM using all available visits | 152 | -0.74 (-1.11, -0.36) | <0.001 |
| Pruritus NRS | LOCF sensitivity ANCOVA | 152 | -1.00 (-1.24, -0.76) | <0.001 |
| Pruritus NRS | BOCF sensitivity ANCOVA | 152 | -0.89 (-1.22, -0.55) | <0.001 |
| Pruritus NRS | Multiple imputation ANCOVA, m=50 | 152 | -0.96 (-1.17, -0.75) | <0.001 |
| DLQI | Complete-case ANCOVA | 124 | -2.86 (-3.47, -2.26) | <0.001 |
| DLQI | MMRM-style LMM using all available visits | 152 | -2.15 (-3.27, -1.04) | <0.001 |
| DLQI | LOCF sensitivity ANCOVA | 152 | -2.85 (-3.49, -2.21) | <0.001 |
| DLQI | BOCF sensitivity ANCOVA | 152 | -2.68 (-3.61, -1.76) | <0.001 |
| DLQI | Multiple imputation ANCOVA, m=50 | 152 | -2.76 (-3.36, -2.16) | <0.001 |

*For MMRM-style models, the treatment effect represents the estimated week-12 between-group contrast from a mixed model using all available visits.*

**Table S7. Transparent sample-size justification parameters.**

| **Assumption** | **Value** |
| --- | --- |
| Observed pooled SD of EASI change among completers | 2.15 |
| Observed pooled SD of week-12 EASI among completers | 4.16 |
| Expected between-group difference used for justification | 3.0 |
| Alpha level | 0.05 |
| Target power | 0.8 |
| Required n per group using pooled SD of EASI change | 10.0 |
| Required n per group using pooled SD of week-12 EASI | 32.0 |
| Required randomized n per group after attrition inflation using EASI change SD | 12.0 |
| Actual randomized n per group | 76.0 |

*This table provides a transparent post hoc sample-size justification using observed EASI variability.*

**Table S8. Clinically meaningful EASI improvement thresholds.**

| **Endpoint** | **Placebo** | **Qing-Re-Qu-Shi** | **Risk ratio (95% CI)** | **P value** |
| --- | --- | --- | --- | --- |
| Absolute EASI improvement ≥3 | 55/76 (72.4%) | 63/76 (82.9%) | 1.15 (0.96, 1.36) | 0.173 |
| Absolute EASI improvement ≥4 | 47/76 (61.8%) | 62/76 (81.6%) | 1.32 (1.07, 1.62) | 0.011 |
| Absolute EASI improvement ≥6.6 | 14/76 (18.4%) | 55/76 (72.4%) | 3.93 (2.40, 6.43) | <0.001 |
| EASI-50 response | 12/76 (15.8%) | 49/76 (64.5%) | 4.08 (2.37, 7.04) | <0.001 |
| EASI-75 response | 2/76 (2.6%) | 15/76 (19.7%) | 7.50 (1.78, 31.68) | 0.001 |

*Participants without week-12 EASI were counted as not achieving the threshold.*

**Table S9. Adherence by treatment group.**

| **Group** | **n** | **Mean ± SD** | **Median (IQR)** | **n below 80%** | **% below 80%** | **Group comparison P** |
| --- | --- | --- | --- | --- | --- | --- |
| Placebo | 76 | 0.888 ± 0.073 | 0.893 (0.844, 0.942) | 9 | 11.8% | 0.822 |
| Qing-Re-Qu-Shi | 76 | 0.889 ± 0.080 | 0.898 (0.841, 0.948) | 11 | 14.5% |  |

*Adherence was assessed by treatment reconciliation and participant interview.*

**Table S10. Adherence and per-protocol sensitivity analyses.**

| **Endpoint** | **Model** | **n** | **Treatment effect (95% CI)** | **P value** |
| --- | --- | --- | --- | --- |
| EASI | Per-protocol ANCOVA, completed and adherence ≥80% | 109 | -3.84 (-4.66, -3.02) | <0.001 |
| EASI | ANCOVA adjusted for adherence proportion | 124 | -3.76 (-4.53, -3.00) | <0.001 |
| SCORAD | Per-protocol ANCOVA, completed and adherence ≥80% | 109 | -9.70 (-11.69, -7.71) | <0.001 |
| SCORAD | ANCOVA adjusted for adherence proportion | 124 | -9.74 (-11.55, -7.93) | <0.001 |
| POEM | Per-protocol ANCOVA, completed and adherence ≥80% | 109 | -3.45 (-4.17, -2.73) | <0.001 |
| POEM | ANCOVA adjusted for adherence proportion | 124 | -3.39 (-4.06, -2.72) | <0.001 |
| Pruritus NRS | Per-protocol ANCOVA, completed and adherence ≥80% | 109 | -0.96 (-1.19, -0.73) | <0.001 |
| Pruritus NRS | ANCOVA adjusted for adherence proportion | 124 | -0.94 (-1.16, -0.72) | <0.001 |
| DLQI | Per-protocol ANCOVA, completed and adherence ≥80% | 109 | -2.91 (-3.57, -2.25) | <0.001 |
| DLQI | ANCOVA adjusted for adherence proportion | 124 | -2.85 (-3.45, -2.24) | <0.001 |

*Per-protocol analysis included participants completing week 12 with adherence ≥80%.*

**Table S11. Rescue topical therapy data availability.**

| **Variable** | **Placebo** | **Qing-Re-Qu-Shi** | **Risk ratio (95% CI)** | **P value** |
| --- | --- | --- | --- | --- |
| No rescue-therapy column detected | Not available | Not available | Not estimable |  |

*No rescue-therapy frequency column was available in the analysis dataset.*

**Table S12. Microbiome read-depth and alpha-diversity quality summary.**

| **Group** | **Time point** | **n samples** | **Total reads, median (IQR)** | **Count-table depth, median (IQR)** | **Shannon, median (IQR)** | **Observed genera, median (IQR)** |
| --- | --- | --- | --- | --- | --- | --- |
| Placebo | Baseline | 76 | 39508 (32691, 43738) | 39508 (32691, 43738) | 2.738 (2.707, 2.770) | 20 (20, 20) |
| Placebo | Week12 | 60 | 37188 (30646, 40920) | 37188 (30646, 40920) | 2.732 (2.700, 2.767) | 20 (20, 20) |
| Qing-Re-Qu-Shi | Baseline | 76 | 37392 (32131, 42653) | 37392 (32131, 42653) | 2.742 (2.700, 2.761) | 20 (20, 20) |
| Qing-Re-Qu-Shi | Week12 | 64 | 37094 (33314, 42884) | 37094 (33314, 42884) | 2.722 (2.678, 2.762) | 20 (20, 20) |

**Table S13. Taxa prevalence and zero handling summary.**

| **Taxon** | **Total counts** | **Prevalence n** | **Prevalence %** | **Zero %** | **Tested in full-feature analysis** |
| --- | --- | --- | --- | --- | --- |
| bacteroides | 1637249 | 276 | 100 | 0 | True |
| prevotella | 1116119 | 276 | 100 | 0 | True |
| bifidobacterium | 950002 | 276 | 100 | 0 | True |
| faecalibacteriµm | 865964 | 276 | 100 | 0 | True |
| blautia | 690362 | 276 | 100 | 0 | True |
| ruminococcus | 569064 | 276 | 100 | 0 | True |
| roseburia | 566807 | 276 | 100 | 0 | True |
| agathobacter | 510737 | 276 | 100 | 0 | True |
| lachnospiraceae_nk4a136_group | 451634 | 276 | 100 | 0 | True |
| subdoligranulum | 435498 | 276 | 100 | 0 | True |
| alistipes | 366507 | 276 | 100 | 0 | True |
| lactobacillus | 326498 | 276 | 100 | 0 | True |
| collinsella | 302208 | 276 | 100 | 0 | True |
| megamonas | 295030 | 276 | 100 | 0 | True |
| parasutterella | 261106 | 276 | 100 | 0 | True |
| akkermansia | 242128 | 276 | 100 | 0 | True |
| escherichia_shigella | 238139 | 276 | 100 | 0 | True |
| clostridium_sensu_stricto_1 | 222611 | 276 | 100 | 0 | True |
| romboutsia | 205188 | 276 | 100 | 0 | True |
| enterococcus | 171713 | 276 | 100 | 0 | True |

*Zero values were handled using a pseudocount before CLR transformation.*

**Table S14. Shannon diversity linear mixed-effects model.**

| **Term** | **df** | **Statistic** | **Raw P value** | **P value** |
| --- | --- | --- | --- | --- |
| group | 151.84582697413185 | 0.1087816559717705 | 0.741990044962354 | 0.742 |
| timepoint | 136.33280109531384 | 3.080299769850208 | 0.0814922137867416 | 0.081 |
| group:timepoint | 136.33280109387044 | 2.9845925847243358 | 0.0863249724628109 | 0.086 |

**Table S15. Repeated-measures-aware PERMANOVA for Bray-Curtis β diversity.**

| **Term** | **df** | **Sum of squares** | **R²** | **F statistic** | **P value** |
| --- | --- | --- | --- | --- | --- |
| group:timepoint | 1 | 0.0779017217235376 | 0.0161592919239769 | 4.126290586876973 | 0.001 |
| Residual | 244 | 4.606563619391042 | 0.9555476393228496 |  |  |
| Total | 247 | 4.820862330480447 | 1.0 |  |  |

*Permutations were restricted within participant ID. The group-by-time effect was statistically significant but small (R²=0.016).*

**Table S16. PERMDISP analysis.**

| **Test** | **Statistic** | **Raw P value** | **P value** |
| --- | --- | --- | --- |
| PERMDISP by group-time strata | 2.2905929554353914 | 0.085 | 0.085 |

**Table S17. Within-subject Bray-Curtis distance summary.**

| **Group** | **n** | **Median (IQR)** | **Mean ± SD** |
| --- | --- | --- | --- |
| Placebo | 60 | 0.141 (0.128, 0.163) | 0.143 ± 0.026 |
| Qing-Re-Qu-Shi | 64 | 0.146 (0.128, 0.163) | 0.145 ± 0.026 |

**Table S18. Within-subject Bray-Curtis distance model.**

| **Model** | **n** | **Adjusted difference (95% CI)** | **P value** |
| --- | --- | --- | --- |
| Within-subject Bray-Curtis distance from baseline to week 12 | 124 | 0.002 (-0.007, 0.011) | 0.641 |

**Table S19. Full-feature CLR analysis of all tested taxa.**

| **Taxon** | **n** | **Adjusted β (95% CI)** | **P value** | **FDR-adjusted P** | **Direction** |
| --- | --- | --- | --- | --- | --- |
| Blautia | 124 | 0.328 (0.216, 0.441) | <0.001 | <0.001 | Higher week-12 CLR abundance in formula group |
| Bifidobacterium | 124 | 0.319 (0.207, 0.431) | <0.001 | <0.001 | Higher week-12 CLR abundance in formula group |
| Lactobacillus | 124 | 0.350 (0.189, 0.511) | <0.001 | <0.001 | Higher week-12 CLR abundance in formula group |
| Escherichia/Shigella | 124 | -0.350 (-0.524, -0.176) | <0.001 | <0.001 | Lower week-12 CLR abundance in formula group |
| Enterococcus | 124 | -0.512 (-0.788, -0.235) | <0.001 | 0.001 | Lower week-12 CLR abundance in formula group |
| Agathobacter | 124 | 0.206 (0.078, 0.334) | 0.002 | 0.006 | Higher week-12 CLR abundance in formula group |
| Prevotella | 124 | -0.122 (-0.218, -0.026) | 0.013 | 0.037 | Lower week-12 CLR abundance in formula group |
| Collinsella | 124 | -0.202 (-0.370, -0.035) | 0.018 | 0.045 | Lower week-12 CLR abundance in formula group |
| clostridium sensu stricto 1 | 124 | -0.192 (-0.367, -0.016) | 0.032 | 0.072 | Lower week-12 CLR abundance in formula group |
| roseburia | 124 | 0.121 (0.000, 0.241) | 0.049 | 0.086 | Higher week-12 CLR abundance in formula group |
| lachnospiraceae nk4a136 group | 124 | 0.121 (-0.000, 0.243) | 0.051 | 0.086 | Higher week-12 CLR abundance in formula group |
| ruminococcus | 124 | -0.130 (-0.261, 0.001) | 0.052 | 0.086 | Lower week-12 CLR abundance in formula group |
| megamonas | 124 | 0.121 (-0.026, 0.269) | 0.107 | 0.164 | Higher week-12 CLR abundance in formula group |
| akkermansia | 124 | 0.130 (-0.034, 0.294) | 0.119 | 0.169 | Higher week-12 CLR abundance in formula group |
| parasutterella | 124 | -0.085 (-0.247, 0.077) | 0.299 | 0.399 | Lower week-12 CLR abundance in formula group |
| bacteroides | 124 | -0.039 (-0.121, 0.044) | 0.353 | 0.442 | Lower week-12 CLR abundance in formula group |
| romboutsia | 124 | -0.067 (-0.247, 0.114) | 0.468 | 0.551 | Lower week-12 CLR abundance in formula group |
| faecalibacteriµm | 124 | -0.027 (-0.128, 0.074) | 0.598 | 0.665 | Lower week-12 CLR abundance in formula group |
| alistipes | 124 | 0.012 (-0.106, 0.131) | 0.837 | 0.881 | Higher week-12 CLR abundance in formula group |
| subdoligranulum | 124 | 0.005 (-0.130, 0.141) | 0.936 | 0.936 | Higher week-12 CLR abundance in formula group |

*Adjusted β values represent between-group differences in CLR-transformed change from baseline to week 12, adjusted for baseline abundance, age, and sex.*

**Table S20. Full targeted serum-metabolite analysis.**

| **Metabolite** | **n** | **Adjusted β (95% CI)** | **P value** | **FDR-adjusted P** | **Direction** |
| --- | --- | --- | --- | --- | --- |
| Indole-3-aldehyde | 124 | 0.507 (0.439, 0.575) | <0.001 | <0.001 | Higher week-12 log2 concentration in formula group |
| Butyrate | 124 | 0.368 (0.301, 0.434) | <0.001 | <0.001 | Higher week-12 log2 concentration in formula group |
| Indole-3-lactic acid | 124 | 0.369 (0.300, 0.437) | <0.001 | <0.001 | Higher week-12 log2 concentration in formula group |
| Indole-3-propionic acid | 124 | 0.336 (0.265, 0.408) | <0.001 | <0.001 | Higher week-12 log2 concentration in formula group |
| Kynurenine | 124 | -0.260 (-0.329, -0.192) | <0.001 | <0.001 | Lower week-12 log2 concentration in formula group |
| Propionate | 124 | 0.225 (0.131, 0.318) | <0.001 | <0.001 | Higher week-12 log2 concentration in formula group |
| Kynurenine/tryptophan ratio | 124 | -0.307 (-0.436, -0.178) | <0.001 | <0.001 | Lower week-12 log2 concentration in formula group |
| Acetate | 124 | 0.160 (0.077, 0.243) | <0.001 | <0.001 | Higher week-12 log2 concentration in formula group |
| Tryptophan | 124 | 0.036 (-0.062, 0.134) | 0.470 | 0.470 | Higher week-12 log2 concentration in formula group |

*Adjusted β values represent between-group differences in log2-transformed change from baseline to week 12, adjusted for baseline metabolite level, age, and sex.*

**Table S21. QRQS-arm responder versus non-responder omics comparisons.**

| **Response definition** | **Feature type** | **Feature** | **n** | **Responder n** | **Non-responder n** | **Estimate (95% CI)** | **P value** | **FDR-adjusted P** |
| --- | --- | --- | --- | --- | --- | --- | --- | --- |
| EASI-50 responder within Qing-Re-Qu-Shi completers | Serum metabolite log2 | kynurenine_nmol_g | 54 | 40 | 14 | -0.121 (-0.248, 0.006) | 0.062 | 0.557 |
| EASI-50 responder within Qing-Re-Qu-Shi completers | Serum metabolite log2 | butyrate_µmol_g | 54 | 40 | 14 | 0.039 (-0.069, 0.146) | 0.471 | 0.707 |
| EASI-50 responder within Qing-Re-Qu-Shi completers | Serum metabolite log2 | indole_3_aldehyde_nmol_g | 54 | 40 | 14 | 0.053 (-0.092, 0.197) | 0.467 | 0.707 |
| EASI-50 responder within Qing-Re-Qu-Shi completers | Serum metabolite log2 | indole_3_lactic_acid_nmol_g | 54 | 40 | 14 | -0.057 (-0.184, 0.069) | 0.366 | 0.707 |
| EASI-50 responder within Qing-Re-Qu-Shi completers | Serum metabolite log2 | kynurenine_tryptophan_ratio | 54 | 40 | 14 | -0.151 (-0.393, 0.090) | 0.214 | 0.707 |
| EASI-50 responder within Qing-Re-Qu-Shi completers | Serum metabolite log2 | propionate_µmol_g | 54 | 40 | 14 | 0.106 (-0.078, 0.290) | 0.251 | 0.707 |
| EASI-50 responder within Qing-Re-Qu-Shi completers | Serum metabolite log2 | acetate_µmol_g | 54 | 40 | 14 | -0.034 (-0.188, 0.119) | 0.655 | 0.74 |
| EASI-50 responder within Qing-Re-Qu-Shi completers | Serum metabolite log2 | indole_3_propionic_acid_nmol_g | 54 | 40 | 14 | -0.029 (-0.185, 0.127) | 0.711 | 0.74 |
| EASI-50 responder within Qing-Re-Qu-Shi completers | Serum metabolite log2 | tryptophan_nmol_g | 54 | 40 | 14 | 0.026 (-0.132, 0.185) | 0.74 | 0.74 |
| EASI-50 responder within Qing-Re-Qu-Shi completers | Taxon CLR | bacteroides | 54 | 40 | 14 | -0.166 (-0.306, -0.026) | 0.021 | 0.312 |
| EASI-50 responder within Qing-Re-Qu-Shi completers | Taxon CLR | subdoligranulum | 54 | 40 | 14 | 0.247 (0.023, 0.471) | 0.031 | 0.312 |
| EASI-50 responder within Qing-Re-Qu-Shi completers | Taxon CLR | alistipes | 54 | 40 | 14 | 0.203 (-0.013, 0.419) | 0.065 | 0.433 |
| EASI-50 responder within Qing-Re-Qu-Shi completers | Taxon CLR | escherichia_shigella | 54 | 40 | 14 | -0.241 (-0.548, 0.066) | 0.121 | 0.606 |
| EASI-50 responder within Qing-Re-Qu-Shi completers | Taxon CLR | akkermansia | 54 | 40 | 14 | 0.143 (-0.121, 0.406) | 0.282 | 0.667 |
| EASI-50 responder within Qing-Re-Qu-Shi completers | Taxon CLR | clostridium_sensu_stricto_1 | 54 | 40 | 14 | -0.216 (-0.592, 0.160) | 0.255 | 0.667 |
| EASI-50 responder within Qing-Re-Qu-Shi completers | Taxon CLR | collinsella | 54 | 40 | 14 | -0.200 (-0.512, 0.111) | 0.202 | 0.667 |
| EASI-50 responder within Qing-Re-Qu-Shi completers | Taxon CLR | lachnospiraceae_nk4a136_group | 54 | 40 | 14 | -0.123 (-0.361, 0.115) | 0.305 | 0.667 |
| EASI-50 responder within Qing-Re-Qu-Shi completers | Taxon CLR | lactobacillus | 54 | 40 | 14 | 0.129 (-0.110, 0.368) | 0.284 | 0.667 |
| EASI-50 responder within Qing-Re-Qu-Shi completers | Taxon CLR | megamonas | 54 | 40 | 14 | 0.122 (-0.129, 0.373) | 0.334 | 0.667 |
| EASI-50 responder within Qing-Re-Qu-Shi completers | Taxon CLR | agathobacter | 54 | 40 | 14 | -0.077 (-0.279, 0.125) | 0.448 | 0.814 |
| EASI-50 responder within Qing-Re-Qu-Shi completers | Taxon CLR | blautia | 54 | 40 | 14 | 0.053 (-0.148, 0.254) | 0.601 | 0.839 |
| EASI-50 responder within Qing-Re-Qu-Shi completers | Taxon CLR | faecalibacteriµm | 54 | 40 | 14 | 0.053 (-0.125, 0.230) | 0.553 | 0.839 |
| EASI-50 responder within Qing-Re-Qu-Shi completers | Taxon CLR | parasutterella | 54 | 40 | 14 | 0.056 (-0.206, 0.317) | 0.671 | 0.839 |
| EASI-50 responder within Qing-Re-Qu-Shi completers | Taxon CLR | prevotella | 54 | 40 | 14 | 0.056 (-0.140, 0.251) | 0.568 | 0.839 |
| EASI-50 responder within Qing-Re-Qu-Shi completers | Taxon CLR | roseburia | 54 | 40 | 14 | -0.050 (-0.280, 0.181) | 0.668 | 0.839 |
| EASI-50 responder within Qing-Re-Qu-Shi completers | Taxon CLR | bifidobacterium | 54 | 40 | 14 | -0.021 (-0.239, 0.196) | 0.844 | 0.951 |
| EASI-50 responder within Qing-Re-Qu-Shi completers | Taxon CLR | romboutsia | 54 | 40 | 14 | -0.030 (-0.359, 0.300) | 0.856 | 0.951 |
| EASI-50 responder within Qing-Re-Qu-Shi completers | Taxon CLR | enterococcus | 54 | 40 | 14 | 0.014 (-0.618, 0.645) | 0.965 | 0.969 |
| EASI-50 responder within Qing-Re-Qu-Shi completers | Taxon CLR | ruminococcus | 54 | 40 | 14 | -0.005 (-0.266, 0.256) | 0.969 | 0.969 |
| EASI-75 responder within Qing-Re-Qu-Shi completers | Serum metabolite log2 | indole_3_lactic_acid_nmol_g | 54 | 12 | 42 | 0.112 (-0.013, 0.237) | 0.077 | 0.627 |
| EASI-75 responder within Qing-Re-Qu-Shi completers | Serum metabolite log2 | kynurenine_nmol_g | 54 | 12 | 42 | -0.096 (-0.225, 0.032) | 0.139 | 0.627 |
| EASI-75 responder within Qing-Re-Qu-Shi completers | Serum metabolite log2 | acetate_µmol_g | 54 | 12 | 42 | 0.037 (-0.121, 0.195) | 0.637 | 0.876 |
| EASI-75 responder within Qing-Re-Qu-Shi completers | Serum metabolite log2 | butyrate_µmol_g | 54 | 12 | 42 | 0.030 (-0.078, 0.137) | 0.578 | 0.876 |
| EASI-75 responder within Qing-Re-Qu-Shi completers | Serum metabolite log2 | indole_3_propionic_acid_nmol_g | 54 | 12 | 42 | -0.047 (-0.206, 0.112) | 0.556 | 0.876 |
| EASI-75 responder within Qing-Re-Qu-Shi completers | Serum metabolite log2 | kynurenine_tryptophan_ratio | 54 | 12 | 42 | -0.051 (-0.301, 0.199) | 0.681 | 0.876 |
| EASI-75 responder within Qing-Re-Qu-Shi completers | Serum metabolite log2 | tryptophan_nmol_g | 54 | 12 | 42 | -0.048 (-0.211, 0.114) | 0.554 | 0.876 |
| EASI-75 responder within Qing-Re-Qu-Shi completers | Serum metabolite log2 | indole_3_aldehyde_nmol_g | 54 | 12 | 42 | 0.018 (-0.131, 0.166) | 0.812 | 0.913 |
| EASI-75 responder within Qing-Re-Qu-Shi completers | Serum metabolite log2 | propionate_µmol_g | 54 | 12 | 42 | 0.009 (-0.182, 0.200) | 0.922 | 0.922 |
| EASI-75 responder within Qing-Re-Qu-Shi completers | Taxon CLR | subdoligranulum | 54 | 12 | 42 | 0.273 (0.046, 0.499) | 0.019 | 0.388 |
| EASI-75 responder within Qing-Re-Qu-Shi completers | Taxon CLR | agathobacter | 54 | 12 | 42 | -0.028 (-0.232, 0.176) | 0.785 | 0.872 |
| EASI-75 responder within Qing-Re-Qu-Shi completers | Taxon CLR | akkermansia | 54 | 12 | 42 | 0.061 (-0.214, 0.336) | 0.658 | 0.872 |
| EASI-75 responder within Qing-Re-Qu-Shi completers | Taxon CLR | alistipes | 54 | 12 | 42 | 0.085 (-0.142, 0.312) | 0.457 | 0.872 |
| EASI-75 responder within Qing-Re-Qu-Shi completers | Taxon CLR | bacteroides | 54 | 12 | 42 | -0.040 (-0.192, 0.112) | 0.597 | 0.872 |
| EASI-75 responder within Qing-Re-Qu-Shi completers | Taxon CLR | bifidobacterium | 54 | 12 | 42 | -0.101 (-0.320, 0.119) | 0.36 | 0.872 |
| EASI-75 responder within Qing-Re-Qu-Shi completers | Taxon CLR | blautia | 54 | 12 | 42 | 0.031 (-0.177, 0.238) | 0.768 | 0.872 |
| EASI-75 responder within Qing-Re-Qu-Shi completers | Taxon CLR | collinsella | 54 | 12 | 42 | -0.139 (-0.453, 0.174) | 0.377 | 0.872 |
| EASI-75 responder within Qing-Re-Qu-Shi completers | Taxon CLR | enterococcus | 54 | 12 | 42 | 0.101 (-0.547, 0.749) | 0.755 | 0.872 |
| EASI-75 responder within Qing-Re-Qu-Shi completers | Taxon CLR | escherichia_shigella | 54 | 12 | 42 | -0.098 (-0.419, 0.222) | 0.541 | 0.872 |
| EASI-75 responder within Qing-Re-Qu-Shi completers | Taxon CLR | faecalibacteriµm | 54 | 12 | 42 | 0.064 (-0.119, 0.248) | 0.485 | 0.872 |
| EASI-75 responder within Qing-Re-Qu-Shi completers | Taxon CLR | lachnospiraceae_nk4a136_group | 54 | 12 | 42 | -0.112 (-0.356, 0.131) | 0.359 | 0.872 |
| EASI-75 responder within Qing-Re-Qu-Shi completers | Taxon CLR | lactobacillus | 54 | 12 | 42 | -0.050 (-0.302, 0.203) | 0.694 | 0.872 |
| EASI-75 responder within Qing-Re-Qu-Shi completers | Taxon CLR | megamonas | 54 | 12 | 42 | 0.171 (-0.084, 0.426) | 0.184 | 0.872 |
| EASI-75 responder within Qing-Re-Qu-Shi completers | Taxon CLR | parasutterella | 54 | 12 | 42 | 0.162 (-0.101, 0.426) | 0.222 | 0.872 |
| EASI-75 responder within Qing-Re-Qu-Shi completers | Taxon CLR | prevotella | 54 | 12 | 42 | -0.032 (-0.232, 0.169) | 0.751 | 0.872 |
| EASI-75 responder within Qing-Re-Qu-Shi completers | Taxon CLR | romboutsia | 54 | 12 | 42 | -0.260 (-0.591, 0.071) | 0.121 | 0.872 |
| EASI-75 responder within Qing-Re-Qu-Shi completers | Taxon CLR | roseburia | 54 | 12 | 42 | -0.166 (-0.402, 0.069) | 0.162 | 0.872 |
| EASI-75 responder within Qing-Re-Qu-Shi completers | Taxon CLR | clostridium_sensu_stricto_1 | 54 | 12 | 42 | -0.037 (-0.426, 0.352) | 0.848 | 0.893 |
| EASI-75 responder within Qing-Re-Qu-Shi completers | Taxon CLR | ruminococcus | 54 | 12 | 42 | 0.016 (-0.253, 0.285) | 0.906 | 0.906 |

*Exploratory within-arm comparisons did not identify robust FDR-significant differences.*

**Table S22. Selected taxa-indole serum-metabolite correlations.**

| **Taxon** | **Indole metabolite** | **n** | **Spearman ρ** | **P value** | **FDR-adjusted P** |
| --- | --- | --- | --- | --- | --- |
| bifidobacterium | indole_3_aldehyde_nmol_g | 124 | 0.401 | <0.001 | <0.001 |
| blautia | indole_3_lactic_acid_nmol_g | 124 | 0.377 | <0.001 | <0.001 |
| bifidobacterium | indole_3_propionic_acid_nmol_g | 124 | 0.372 | <0.001 | <0.001 |
| lactobacillus | indole_3_aldehyde_nmol_g | 124 | 0.368 | <0.001 | <0.001 |
| blautia | indole_3_aldehyde_nmol_g | 124 | 0.365 | <0.001 | <0.001 |
| bifidobacterium | indole_3_lactic_acid_nmol_g | 124 | 0.358 | <0.001 | <0.001 |
| blautia | indole_3_propionic_acid_nmol_g | 124 | 0.329 | <0.001 | 0.001 |
| agathobacter | indole_3_aldehyde_nmol_g | 124 | 0.329 | <0.001 | 0.001 |
| escherichia_shigella | indole_3_lactic_acid_nmol_g | 124 | -0.32 | <0.001 | 0.002 |
| lactobacillus | indole_3_propionic_acid_nmol_g | 124 | 0.306 | <0.001 | 0.003 |
| enterococcus | indole_3_aldehyde_nmol_g | 124 | -0.303 | <0.001 | 0.003 |
| agathobacter | indole_3_propionic_acid_nmol_g | 124 | 0.293 | <0.001 | 0.005 |
| escherichia_shigella | indole_3_aldehyde_nmol_g | 124 | -0.291 | 0.001 | 0.005 |
| escherichia_shigella | indole_3_propionic_acid_nmol_g | 124 | -0.29 | 0.001 | 0.005 |
| enterococcus | indole_3_lactic_acid_nmol_g | 124 | -0.288 | 0.001 | 0.005 |
| agathobacter | indole_3_lactic_acid_nmol_g | 124 | 0.281 | 0.002 | 0.006 |
| lactobacillus | indole_3_lactic_acid_nmol_g | 124 | 0.279 | 0.002 | 0.006 |
| collinsella | indole_3_aldehyde_nmol_g | 124 | -0.236 | 0.008 | 0.028 |
| lachnospiraceae_nk4a136_group | indole_3_aldehyde_nmol_g | 124 | 0.224 | 0.012 | 0.039 |
| roseburia | indole_3_aldehyde_nmol_g | 124 | 0.208 | 0.021 | 0.062 |
| roseburia | indole_3_lactic_acid_nmol_g | 124 | 0.202 | 0.024 | 0.070 |
| collinsella | indole_3_propionic_acid_nmol_g | 124 | -0.197 | 0.029 | 0.078 |
| clostridium_sensu_stricto_1 | indole_3_propionic_acid_nmol_g | 124 | -0.192 | 0.033 | 0.086 |
| ruminococcus | indole_3_propionic_acid_nmol_g | 124 | -0.19 | 0.035 | 0.087 |
| roseburia | indole_3_propionic_acid_nmol_g | 124 | 0.188 | 0.036 | 0.087 |
| megamonas | indole_3_lactic_acid_nmol_g | 124 | 0.166 | 0.065 | 0.149 |
| parasutterella | indole_3_aldehyde_nmol_g | 124 | -0.155 | 0.085 | 0.189 |
| ruminococcus | indole_3_aldehyde_nmol_g | 124 | -0.149 | 0.099 | 0.211 |
| ruminococcus | indole_3_lactic_acid_nmol_g | 124 | -0.147 | 0.104 | 0.214 |
| enterococcus | indole_3_propionic_acid_nmol_g | 124 | -0.143 | 0.114 | 0.226 |

*Spearman correlations were used to assess cross-domain concordance; these associations do not prove direct metabolite production by the correlated taxa.*

**Table S23. Selected cross-domain Spearman correlations with clinical improvement.**

| **Outcome** | **Feature type** | **Feature** | **n** | **Spearman ρ** | **P value** | **FDR-adjusted P** |
| --- | --- | --- | --- | --- | --- | --- |
| easi_improvement | Serum metabolite log2 | indole_3_aldehyde_nmol_g | 102 | 0.602 | <0.001 | <0.001 |
| easi_improvement | Serum metabolite log2 | butyrate_µmol_g | 102 | 0.538 | <0.001 | <0.001 |
| easi_improvement | Serum metabolite log2 | indole_3_propionic_acid_nmol_g | 102 | 0.508 | <0.001 | <0.001 |
| easi_improvement | Serum metabolite log2 | indole_3_lactic_acid_nmol_g | 102 | 0.428 | <0.001 | <0.001 |
| easi_improvement | Serum metabolite log2 | kynurenine_nmol_g | 102 | -0.423 | <0.001 | <0.001 |
| easi_improvement | Serum metabolite log2 | kynurenine_tryptophan_ratio | 102 | -0.350 | <0.001 | <0.001 |
| easi_improvement | Serum metabolite log2 | acetate_µmol_g | 102 | 0.199 | 0.044 | 0.057 |
| easi_improvement | Serum metabolite log2 | propionate_µmol_g | 102 | 0.165 | 0.098 | 0.111 |
| easi_improvement | Serum metabolite log2 | tryptophan_nmol_g | 102 | 0.120 | 0.228 | 0.228 |
| easi_improvement | Taxon CLR | bifidobacterium | 102 | 0.413 | <0.001 | <0.001 |
| easi_improvement | Taxon CLR | enterococcus | 102 | -0.342 | <0.001 | 0.004 |
| easi_improvement | Taxon CLR | escherichia_shigella | 102 | -0.322 | <0.001 | 0.006 |
| easi_improvement | Taxon CLR | blautia | 102 | 0.317 | 0.001 | 0.006 |
| easi_improvement | Taxon CLR | lactobacillus | 102 | 0.302 | 0.002 | 0.008 |
| easi_improvement | Taxon CLR | agathobacter | 102 | 0.284 | 0.004 | 0.013 |
| easi_improvement | Taxon CLR | collinsella | 102 | -0.251 | 0.011 | 0.031 |
| easi_improvement | Taxon CLR | lachnospiraceae_nk4a136_group | 102 | 0.170 | 0.088 | 0.220 |
| easi_improvement | Taxon CLR | roseburia | 102 | 0.055 | 0.584 | 0.730 |
| easi_improvement | Taxon CLR | prevotella | 102 | 0.024 | 0.813 | 0.845 |
| pruritus_improvement | Serum metabolite log2 | indole_3_aldehyde_nmol_g | 102 | 0.590 | <0.001 | <0.001 |
| pruritus_improvement | Serum metabolite log2 | butyrate_µmol_g | 102 | 0.560 | <0.001 | <0.001 |
| pruritus_improvement | Serum metabolite log2 | indole_3_lactic_acid_nmol_g | 102 | 0.521 | <0.001 | <0.001 |
| pruritus_improvement | Serum metabolite log2 | indole_3_propionic_acid_nmol_g | 102 | 0.472 | <0.001 | <0.001 |
| pruritus_improvement | Serum metabolite log2 | kynurenine_nmol_g | 102 | -0.375 | <0.001 | <0.001 |
| pruritus_improvement | Serum metabolite log2 | kynurenine_tryptophan_ratio | 102 | -0.346 | <0.001 | <0.001 |
| pruritus_improvement | Serum metabolite log2 | propionate_µmol_g | 102 | 0.223 | 0.024 | 0.031 |
| pruritus_improvement | Serum metabolite log2 | acetate_µmol_g | 102 | 0.195 | 0.049 | 0.056 |
| pruritus_improvement | Serum metabolite log2 | tryptophan_nmol_g | 102 | 0.145 | 0.147 | 0.147 |
| pruritus_improvement | Taxon CLR | bifidobacterium | 102 | 0.317 | 0.001 | 0.023 |
| pruritus_improvement | Taxon CLR | lactobacillus | 102 | 0.297 | 0.002 | 0.024 |
| pruritus_improvement | Taxon CLR | collinsella | 102 | -0.221 | 0.025 | 0.143 |
| pruritus_improvement | Taxon CLR | escherichia_shigella | 102 | -0.217 | 0.029 | 0.143 |
| pruritus_improvement | Taxon CLR | blautia | 102 | 0.199 | 0.045 | 0.181 |
| pruritus_improvement | Taxon CLR | agathobacter | 102 | 0.179 | 0.071 | 0.220 |
| pruritus_improvement | Taxon CLR | enterococcus | 102 | -0.176 | 0.077 | 0.220 |
| pruritus_improvement | Taxon CLR | lachnospiraceae_nk4a136_group | 102 | 0.029 | 0.773 | 0.966 |
| pruritus_improvement | Taxon CLR | prevotella | 102 | -0.012 | 0.906 | 0.989 |
| pruritus_improvement | Taxon CLR | roseburia | 102 | 0.002 | 0.983 | 0.989 |
| scorad_improvement | Serum metabolite log2 | indole_3_aldehyde_nmol_g | 102 | 0.646 | <0.001 | <0.001 |
| scorad_improvement | Serum metabolite log2 | indole_3_propionic_acid_nmol_g | 102 | 0.565 | <0.001 | <0.001 |
| scorad_improvement | Serum metabolite log2 | butyrate_µmol_g | 102 | 0.536 | <0.001 | <0.001 |
| scorad_improvement | Serum metabolite log2 | indole_3_lactic_acid_nmol_g | 102 | 0.512 | <0.001 | <0.001 |
| scorad_improvement | Serum metabolite log2 | kynurenine_nmol_g | 102 | -0.373 | <0.001 | <0.001 |
| scorad_improvement | Serum metabolite log2 | kynurenine_tryptophan_ratio | 102 | -0.336 | <0.001 | <0.001 |
| scorad_improvement | Serum metabolite log2 | acetate_µmol_g | 102 | 0.247 | 0.012 | 0.016 |
| scorad_improvement | Serum metabolite log2 | propionate_µmol_g | 102 | 0.240 | 0.015 | 0.017 |
| scorad_improvement | Serum metabolite log2 | tryptophan_nmol_g | 102 | 0.115 | 0.250 | 0.250 |
| scorad_improvement | Taxon CLR | bifidobacterium | 102 | 0.344 | <0.001 | 0.005 |
| scorad_improvement | Taxon CLR | escherichia_shigella | 102 | -0.340 | <0.001 | 0.005 |
| scorad_improvement | Taxon CLR | lactobacillus | 102 | 0.284 | 0.004 | 0.025 |
| scorad_improvement | Taxon CLR | agathobacter | 102 | 0.270 | 0.006 | 0.026 |
| scorad_improvement | Taxon CLR | blautia | 102 | 0.267 | 0.007 | 0.026 |
| scorad_improvement | Taxon CLR | enterococcus | 102 | -0.228 | 0.021 | 0.070 |
| scorad_improvement | Taxon CLR | collinsella | 102 | -0.186 | 0.061 | 0.174 |
| scorad_improvement | Taxon CLR | lachnospiraceae_nk4a136_group | 102 | 0.134 | 0.179 | 0.448 |
| scorad_improvement | Taxon CLR | prevotella | 102 | 0.017 | 0.868 | 0.986 |
| scorad_improvement | Taxon CLR | roseburia | 102 | -0.002 | 0.986 | 0.986 |

*Analyses included 102 participants with paired fecal 16S data and paired serum metabolomics data and observed week-12 clinical outcomes.*

**Table S24. Exploratory mediation diagnostics.**

| **Feature type** | **Feature** | **n** | **Indirect effect (95% CI)** | **Direct effect** | **Total effect** | **Proportion mediated** | **Diagnostic** |
| --- | --- | --- | --- | --- | --- | --- | --- |
| Taxon CLR | bifidobacterium | 102 | -0.182 (-0.570, 0.231) | -3.564 | -3.738 | 0.049 | indirect CI crosses zero; limited complete-case sample for mediation |
| Taxon CLR | lactobacillus | 102 | -0.272 (-0.606, 0.067) | -3.469 | -3.738 | 0.073 | indirect CI crosses zero; limited complete-case sample for mediation |
| Taxon CLR | blautia | 102 | -0.083 (-0.602, 0.431) | -3.65 | -3.738 | 0.022 | indirect CI crosses zero; limited complete-case sample for mediation |
| Taxon CLR | agathobacter | 102 | 0.016 (-0.169, 0.228) | -3.752 | -3.738 | -0.004 | indirect CI crosses zero; limited complete-case sample for mediation |
| Serum metabolite log2 | butyrate_µmol_g | 102 | 0.106 (-0.447, 0.575) | -3.855 | -3.738 | -0.028 | indirect CI crosses zero; limited complete-case sample for mediation |
| Serum metabolite log2 | indole_3_aldehyde_nmol_g | 102 | -0.347 (-1.098, 0.286) | -3.372 | -3.738 | 0.093 | indirect CI crosses zero; limited complete-case sample for mediation |
| Serum metabolite log2 | indole_3_lactic_acid_nmol_g | 102 | 0.306 (-0.236, 0.825) | -4.044 | -3.738 | -0.082 | indirect CI crosses zero; limited complete-case sample for mediation |
| Serum metabolite log2 | indole_3_propionic_acid_nmol_g | 102 | 0.006 (-0.321, 0.379) | -3.745 | -3.738 | -0.002 | indirect CI crosses zero; limited complete-case sample for mediation |
| Serum metabolite log2 | kynurenine_nmol_g | 102 | -0.054 (-0.492, 0.364) | -3.686 | -3.738 | 0.014 | indirect CI crosses zero; limited complete-case sample for mediation |
| Serum metabolite log2 | kynurenine_tryptophan_ratio | 102 | -0.119 (-0.495, 0.273) | -3.624 | -3.738 | 0.032 | indirect CI crosses zero; limited complete-case sample for mediation |

*Indirect-effect confidence intervals crossed zero; results were therefore not interpreted as evidence of mediation.*

**Table S25. Completion and withdrawal reasons.**

| **Withdrawal reason** | **Placebo** | **Qing-Re-Qu-Shi** |
| --- | --- | --- |
| Completed | 60 (78.9%) | 64 (84.2%) |
| Lost to follow-up | 4 (5.3%) | 2 (2.6%) |
| Perceived insufficient benefit | 4 (5.3%) | 3 (3.9%) |
| Poor adherence | 4 (5.3%) | 6 (7.9%) |
| Withdrew consent | 4 (5.3%) | 1 (1.3%) |

**Figure S1. Forest plot of all tested taxa in full-feature CLR analysis.**

**Figure S2. Forest plot of all targeted serum metabolites in log2-transformed ANCOVA analysis.**
